# Impaired spatial coding and neuronal hyperactivity in the medial entorhinal cortex of aged APP knock-in mice

**DOI:** 10.1016/j.celrep.2026.117505

**Published:** 2026-06-06

**Authors:** Gustavo A. Rodriguez, Andrew Aoun, Eva F. Rothenberg, C. Oliver Shetler, Lorenzo Posani, Srujan V. Vajram, Thomas Tedesco, Anurag Sharma, Stefano Fusi, S. Abid Hussaini

**Affiliations:** 1 Taub Institute for Research on Alzheimer’s Disease and the Aging Brain, Columbia University Irving Medical Center, New York, NY 10032, USA; 2 Burke Neurological Institute, White Plains, NY 10605, USA; 3 Center for Neural Science, New York University, New York, NY 10003, USA; 4 Department of Neuroscience, Columbia University Irving Medical Center, New York, NY 10027, USA; 5 Center for Theoretical Neuroscience, Columbia University, New York, NY 10027, USA; 6 Zuckerman Mind Brain Behavior Institute, Columbia University, New York, NY 10027, USA; 7 Kavli Institute for Brain Science, Columbia University, New York, NY 10027, USA; 8 Department of Pathology and Cell Biology, Columbia University Irving Medical Center, New York, NY 10032, USA; 9 Lead contact

## Abstract

Advanced amyloid beta (A**β**) pathology is associated with aberrant neuronal network activity and cognitive impairment in preclinical Alzheimer’s disease (AD) models. Here, we assess A**β** pathology’s impact on spatial information processing in the medial entorhinal cortex (MEC) of 18-month *App*^*NL-G-F/NL-G-F*^ knock-in (APP KI) mice during exploration of open field arenas. Spatial information scores are decreased in APP KI MEC neurons versus age-matched controls. Border cell firing preferences are unstable across sessions and grid cell spatial periodicity is disrupted. Ratemap stability analysis using the Earth Mover’s Distance indicates increased instability in spatially tuned APP KI neurons. Spatial decoding analysis indicates deficits in position and speed coding in APP KI mice across all comparisons. Additionally, APP KI mice display a mild hyperactive phenotype driven by narrow-spiking putative interneurons. These findings tie A**β**-associated dysregulation in neuronal firing to disruptions in spatial information processing that may underlie cognitive deficits associated with AD.

## In brief

Using the Earth Mover’s Distance and machine learning techniques, Rodriguez et al. show increased instability in spatial representations and impaired spatial information coding in the medial entorhinal cortex (MEC) of aged amyloid knock-in mice. They also report mild hyperactivity in MEC that is driven by hyperactivity in putative interneurons.

## Graphical Abstract

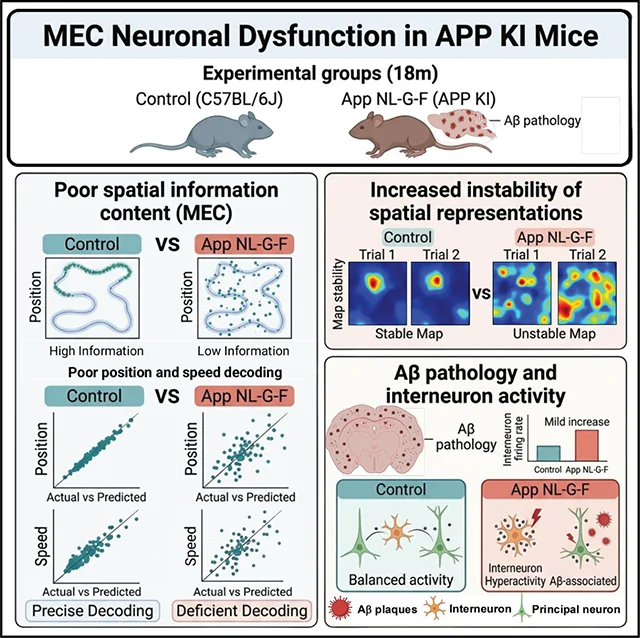

## INTRODUCTION

The entorhinal cortex (EC) and hippocampus play pivotal roles in the processing and integration of spatial information within the mammalian brain.^1–4^ Spatially tuned neurons such as grid, border, head-direction, and place cells exhibit distinctive firing characteristics during active exploration. These cells primarily encode spatial information in a “world-centered” allocentric reference frame, which supports the formation of a stable, objective spatial framework. However, the EC also contains neurons that exhibit spatial tuning in a “self-centered” egocentric reference frame, reflecting spatial relationships relative to one’s position or orientation.^5–7^ Collectively, these cells are thought to contribute to the formation of a cognitive map of space through the utilization of allocentric and egocentric coding schemes.^8–10^ Notably, the EC is also a principal site of aggregated pathology and neuronal loss observed in Alzheimer’s disease (AD), a debilitating neurodegenerative disease characterized symptomatically by a progressive and irreversible decline in cognitive function.^11–14^ Two pathological hallmarks of AD in the brain include the accumulation of hyperphosphorylated, misfolded tau proteins into neurofibrillary tangles (NFT), along with the deposition of amyloid beta (Aβ) into extracellular plaques.^15,16^ Alterations in navigational strategies and reduced grid-cell-like representations in EC have been reported in young adults at genetic risk for AD (*APOE-*ε*4* carriers) and in healthy, aged human subjects.^17,18^ Early pathological conditions during AD progression may therefore compromise spatial coding mechanisms within the EC and hippocampus, manifesting as deficits in path integration and increased spatial disorientation.^19–21^

*In vivo* single-unit recordings in rodent models of AD pathology have yielded important insights into spatial cell dysfunction and its contributions to spatial learning and memory deficits.^22^ Grid-cell representations in the medial EC (MEC) are severely disrupted in mouse models of both Aβ pathology^23–25^ and tau pathology.^26,27^ In the hippocampus, Aβ and tau pathology reduce spatial coding in place cells and impair place field remapping in response to environmental context changes.^23,28–30^ Yet beyond place and grid cells, pathology likely disrupts other spatial cell types, including border, speed, and head-direction cells,^24,25^ as well as nonperiodic spatial cells^31–33^ and non-spatial cells that contribute to spatial coding.^32,34,35^ Aβ and tau pathology may also destabilize spatial coding during exploration of novel and familiar environments. It is therefore critical to examine both the quality and stability of spatial coding in mice with AD pathology, regardless of information quality, to better understand the circuit mechanisms underlying cognitive impairment.

Functional magnetic resonance imaging (fMRI) studies have shown that individuals at risk for AD (e.g., amnestic mild cognitive impairment [aMCI], *APOE-*ε*4* carriers),^36–40^ as well as presymptomatic carriers of familial AD (FAD) mutations,^41^ exhibit pronounced hyperactivity in the hippocampus during task-related encoding. Coupled with an increased prevalence of unprovoked seizures in people with AD and a faster rate of cognitive decline,^42–45^ overexcitation in large networks of neurons may be a clinically relevant feature of AD.^46,47^ Preclinical studies in rodent models of AD pathology have shown that increased neuronal activity plays a key role in both Aβ accumulation^48–50^ and accelerated tau pathology *in vivo*.^51–53^ Moreover, increased seizure susceptibility, neuronal hyperactivity, and network dysfunction have been reported in several rodent models of Aβ pathology.^54–66^ Aβ-associated hyperactivity may indirectly impact spatial coding in the EC and hippocampus by creating a dysfunctional network environment, whereby spatial and non-spatial cells are affected. Alternatively, elevated levels of Aβ pathology (e.g., soluble Aβ species) may directly interact with spatial cells in these regions, disturbing cell firing patterns and distorting spatial tuning properties.

While much is known about the pathophysiology of Aβ and tau progression in the brain, a comprehensive understanding of the mechanisms underlying pathology-associated dysfunction in spatial coding remains incomplete. In this study, we show that advanced Aβ pathology in the brain is associated with impaired spatial information coding in 18-month *App*^*NL-G-F/NL-G-F*^ knock-in (amyloid precursor protein [APP] KI) mice during exploration of novel and familiar contextual environments. Specifically, we show that MEC neurons in aged APP KI mice exhibit decreased spatial information content and impaired firing stability in our paradigm, in addition to poor position and speed coding relative to neurons in age-matched control mice. We also found a mild hyperactivity phenotype in the MEC of APP KI mice. Altogether, these results provide evidence that advanced Aβ pathology in aged mice disturbs spatial information coding in MEC during exploration of novel and familiar environments.

## RESULTS

### MEC neurons in aged APP KI mice exhibit poor spatial information content in familiar and novel contexts

APP KI mice express humanized mutant APP at physiological levels, leading to aggressive age-associated amyloidosis and Aβ plaque formation in the brain beginning as early as 3–4 months.^67,68^ At 6–13 months, when Aβ pathology is substantial, these mice show deficits in context discrimination, spatial learning and memory, and impaired remapping in CA1 place cells and MEC grid cells,^23,56,57,68,69^ collectively implicating Aβ pathology in spatial cell dysfunction and cognitive impairment. Yet the quality of spatial information coding in these mice, and whether it remains stable over time or across familiar and novel environments, remains largely unexplored.

**Figure 1.**
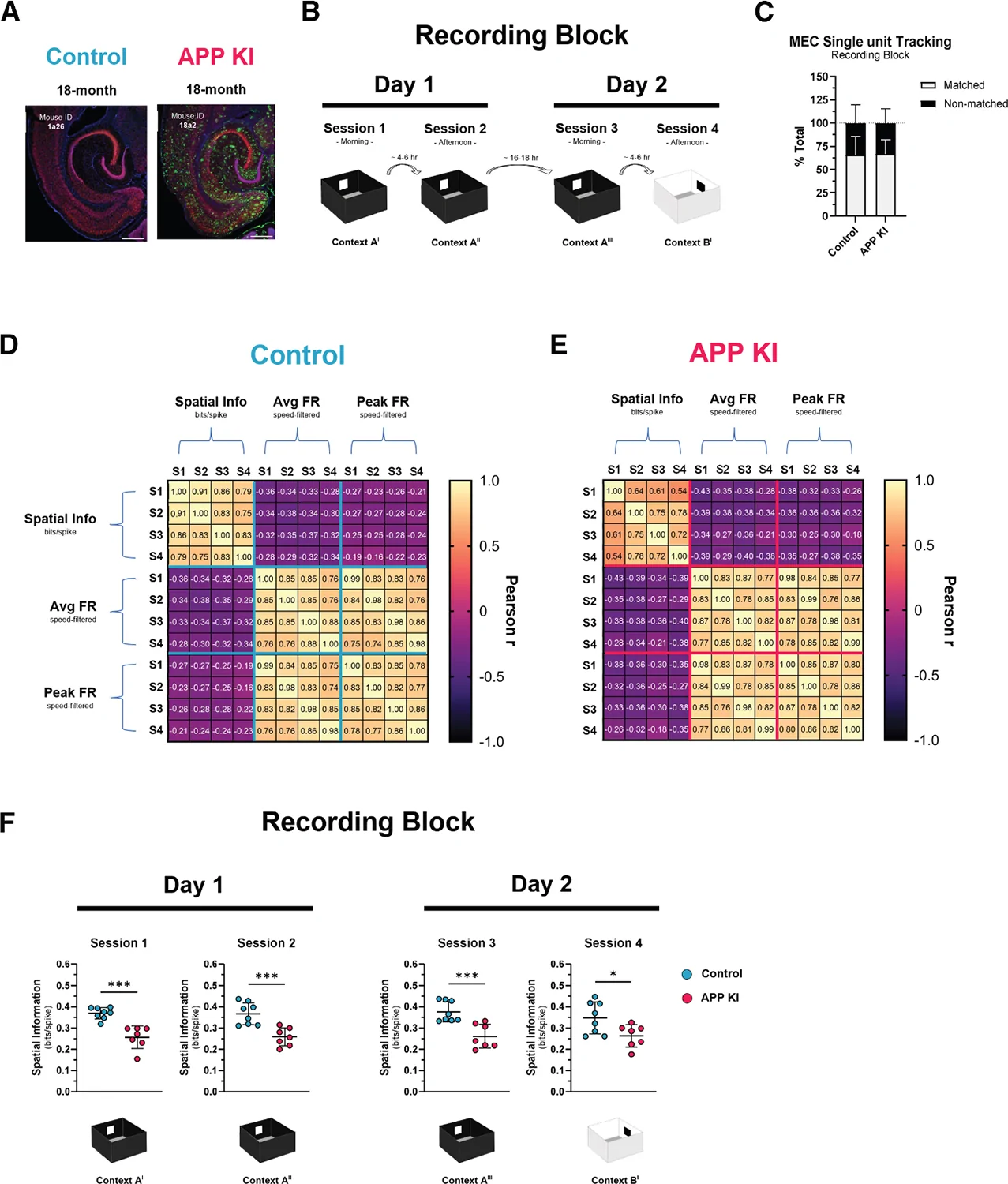
Aged *APP*^*NL-G-F/NL-G-F*^ mice exhibit impaired spatial information processing in the medial entorhinal cortex (A) Immunofluorescence in horizontal brain sections shows significant amyloid beta (Aβ) pathology throughout the entorhinal cortex and hippocampus of 18-month *App* ^*NL-G-F/NL-G-F* ^ (APP KI) mice, absent in age-matched C57BL/6J controls. Aβ, green; NeuN, red; Hoechst, blue. Scale bars, 500 μm. (B) Recording paradigm: session 1, A^I^ (novel); sessions 2–3, context A^II^-A^III^ (familiar); session 4, context B^I^ (Novel). Superscripts indicate exposure number. (C) Proportion of matched vs. non-matched units per mouse (gray, matched; black, non-matched. No genotype difference (unpaired *t* test, *t*_(13)_ = 0.090, *p* = 0.93). (D and E) Cross-session correlation matrices for speed-filtered average firing rate (AVG FR), peak firing rate, and spatial information (SI) score in control and APP KI mice. (F) Mean SI score per mouse, by session. APP KI < control across all sessions (unpaired *t* tests: S1, *t*_(13)_ = 5.37, *p* < 0.001; S2, *t*_(13)_ = 4.42, *p* < 0.001; S3 *t*_(13)_ = 4.26, *p* < 0.001; S4, *t*_(13)_ = 2.45, *p* < 0.05). Plots show mean ± SD. *p < 0.05; **p < 0.01; ***p < 0.001.

To assess the impact of advanced Aβ pathology on stability of spatial information coding over large timescales, we performed *in vivo* MEC recordings in 18-month APP KI mice (*n* = 7) and age-matched C57BL/6J (control) mice (*n* = 8) as they explored square open field arenas across a sequence of recording blocks (Figures 1A and 1B). Recording blocks consisted of four individual sessions spread over two consecutive days, with two sessions per day. Sessions 1 and 2 (S1 and S2) were recorded in the morning and afternoon of day 1; sessions 3 and 4 (S3 and S4) on day 2. The intersession interval (ISI) was 4–6 h for same-day recordings (S1 vs. S2, S3 vs. S4) and 16–18 h for S2 vs. S3. Importantly, arena context remained constant from S1 through S3 and then changed for S4 to promote remapping events and/or spatial map instability. All arena contexts used in these experiments are shown in Figure S1.

Individual MEC neurons were tracked across sessions and classified as “matched” if they reliably appeared in all four sessions, or “non-matched” if they did not appear in all sessions or could not be reliably tracked. In total, 226 matched MEC neurons were recorded and analyzed (control: *n* = 115; APP KI: *n* = 111), while 126 non-matched units were excluded from spatial coding analysis. No significant group differences were found in the number of matched or non-matched units tracked between genotypes (*p* > 0.05) (Figure 1C). To examine task-relevant neuronal activity during active exploration, a speed filter (3–100 cm/s) was applied to remove spiking during immobility (<3 cm/s) and spurious activity (>100 cm/s).

We then examined relationships between speed-filtered average firing rates (AVG FRs), peak firing rates (PK FRs), and spatial information (SI) scores of matched MEC neurons (Figures 1D and 1E). SI scores were calculated using Skaggs’ formula and reflect the degree to which a unit’s firing predicts the animal’s location in the arena.^70,71^ Correlation matrices revealed strong negative relationships between firing rates and SI scores across sessions in both groups, with no discernable genotype differences in AVG FR or PK FR relationships across sessions. However, APP KI MEC neurons showed notably weaker SI score cross-session correlations compared to controls. Consistent with this, APP KI mice exhibited significantly lower SI scores than controls across all four sessions (S1, *p* < 0.001; S2, *p* < 0.001; S3, *p* < 0.001; S4, *p* < 0.05) (Figure 1F). Cross-session correlations of individual MEC unit SI scores further confirmed reduced stability in APP KI mice (Pearson’s r means range: 0.66–0.77) relative to controls (Pearson’s r means range: 0.84–0.92), with significant group differences detected in S1 vs. S2 (*p* < 0.05) and S1 vs. S3 (*p* < 0.05) comparisons but not in other session comparisons (Figure S2).

Speed-filtered (3–100 cm/s) MEC oscillatory activity was also analyzed and compared between genotypes across sessions. No group differences were detected in the percentage power of filtered LFP signals in the delta (1–3 Hz), theta (4–12 Hz), beta (13–20 Hz), low gamma (35–55 Hz), or high gamma (65–120 Hz) frequency bands (all *p* > 0.05) (Figure S3). To ensure locomotor activity did not differ between groups across the two-day recording block, we analyzed position data from freely exploring mice during each session (Figure S4). Total distance traveled, percentage arena coverage, and average speed did not differ significantly between groups across sessions (*p* > 0.05) (Figures S5A–S5D). However, correlation matrices examining relationships between these measures did reveal genotype differences in arena coverage relative to total distance traveled and average speed (Figure S5E). These differences were explained by greater center occupancy and distance traveled in APP KI mice compared to controls (occupancy: zone × genotype, *p* < 0.01; distance: zone × genotype, *p* < 0.05), with post hoc analyses revealing significant group differences in S1 (*p* < 0.01) and S3 (*p* < 0.05) (Figures S5F–S5G). This was further reflected by reduced boundary exploration in APP KI mice in S1 (*p* < 0.01) and S3 (*p* < 0.05).

Together, these data demonstrate that MEC neurons in 18-month APP KI mice exhibit poor spatial information quality during novel environment exploration (S1, context A^I^), which does not improve with repeated exposure to the same environment (S2, context A^II^; S3, context A^III^) or upon exposure to an additional novel environment (S4, context B^I^). Informational stability in APP KI MEC neurons also appeared compromised early in the recording paradigm, as shown by cross-session SI score correlations. These effects could not be attributed to gross locomotor differences, though aged APP KI mice showed a tendency toward increased center exploration relative to controls.

### MEC spatial representations are unstable across familiar and novel contexts in aged APP KI mice

**Figure 2.**
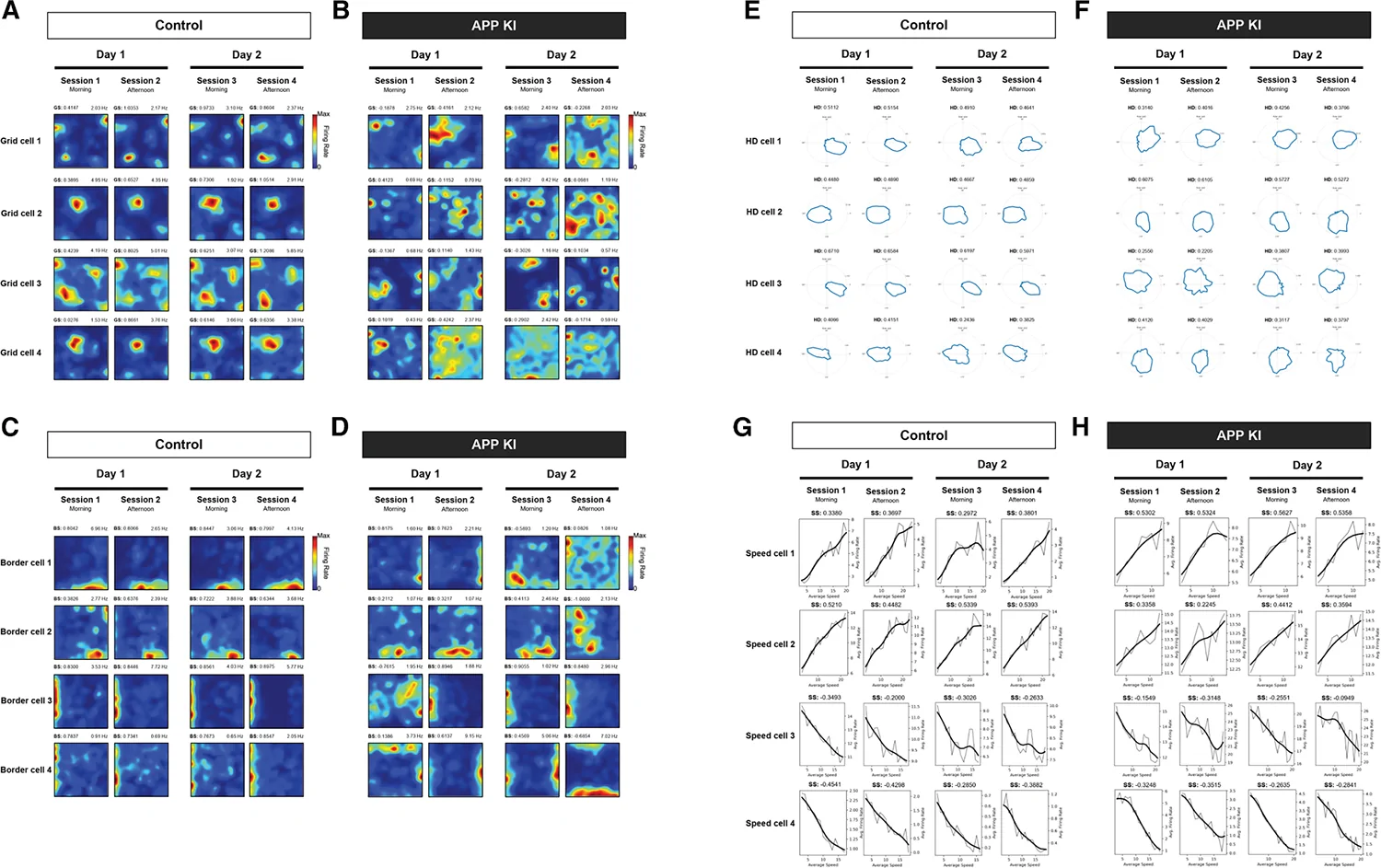
Aged *App*^*NL-G-F/NL-G-F*^ mice exhibit impaired stability of spatial tuning in MEC grid cells and border cells, but not head-direction cells or speed cells Representative MEC cell types. (A and B) Firing rate maps of grid cells in control (A) and APP KI (B) mice across the four-session recording block. (C and D) Firing rate maps of border cells in control (C) and APP KI (D) mice. (E and F) Polar plots of head-direction tuning in control (E) and APP KI (F) mice. (G and H) Tuning curves of speed cells in control (G) and APP KI (H) mice; bold line, smoothed; gray line, non-smoothed. Grid score, border score, HD score, and speed score appear at the top of each plot. Peak ratemap firing rate at top right. Animal IDs, recording block IDs, and cell IDs are as follows: control: 1–25, S29–30, T4C4. 1–28, S14–15, T4C1. 1–34, S12–13, T2C1. 1–28, S14–15, T4C2. APP KI: 1–29, S11–12, T4C1. 1a33, S17–18, T3C1. 1a33, S9–10, T4C1. 1a27, S32–33, T3C2.

To examine the quality and stability of spatial representations of individual MEC neurons across sessions, we identified matched single units exhibiting grid, border, head-direction (HD), and speed tuning in at least one session of the recording block. Spatial scores were calculated per neuron per session, and neurons exceeding the 95^th^–99.5^th^ percentile of a shuffled score distribution were classified as spatial cells (Figures 2 and S6).

A total of 34 MEC neurons (15.04%) passed the criterion for grid cells (Figures S6A and S6B). Control grid cells exhibited hexagonal firing patterns with well-formed, stable fields across all sessions (Figure 2A).^72,73^ In contrast, grid cell tuning was significantly disturbed in APP KI mice, consistent with prior findings in younger knock-in animals.^23^ These nonperiodic, spatially tuned APP KI units displayed scattered firing with dysmorphic fields that were unstable across sessions (Figure 2B). A total of 31 MEC neurons (13.72%) passed the criterion for border cells (Figures S6E and S6F), identified by strong firing fields proximal to environmental boundaries.^74^ Control border cells displayed well-defined, stable fields across sessions (Figure 2C), whereas APP KI border cells showed strong tuning but largely unstable border preference, including rotation and broadening of firing fields and the appearance or disappearance of spatial tuning in both familiar and novel contexts (Figure 2D).

A total of 16 MEC neurons (7.08%) passed the criterion for HD cells (Figures S6I and S6J), which preferentially fired toward a particular direction in polar plots.^75,76^ HD cells were strongly tuned and stable across all sessions in both genotypes, replicating a previous finding in transgenic hAPP/J20 mice (Figures 2E and 2F; Figures S6K and S6L).^24^ A total of 146 MEC neurons (64.60%) passed the criterion for speed cells, classified as either negative (*n* = 68, 30.09%) or positive (*n* = 78, 34.51%) depending on their firing rate relationship with the animal’s speed profile (tuning curve) (Figures S6M, S6N, and S7).^77^ Both negative and positive speed cells were strongly tuned in both groups. Repeated measures analysis detected no significant group differences in averaged spatial scores per cell type across sessions (Figures S6C, S6D, S6G, S6H, S6K, S6L, S6O, S6P, S6Q, and S6R).

**Figure 3.**
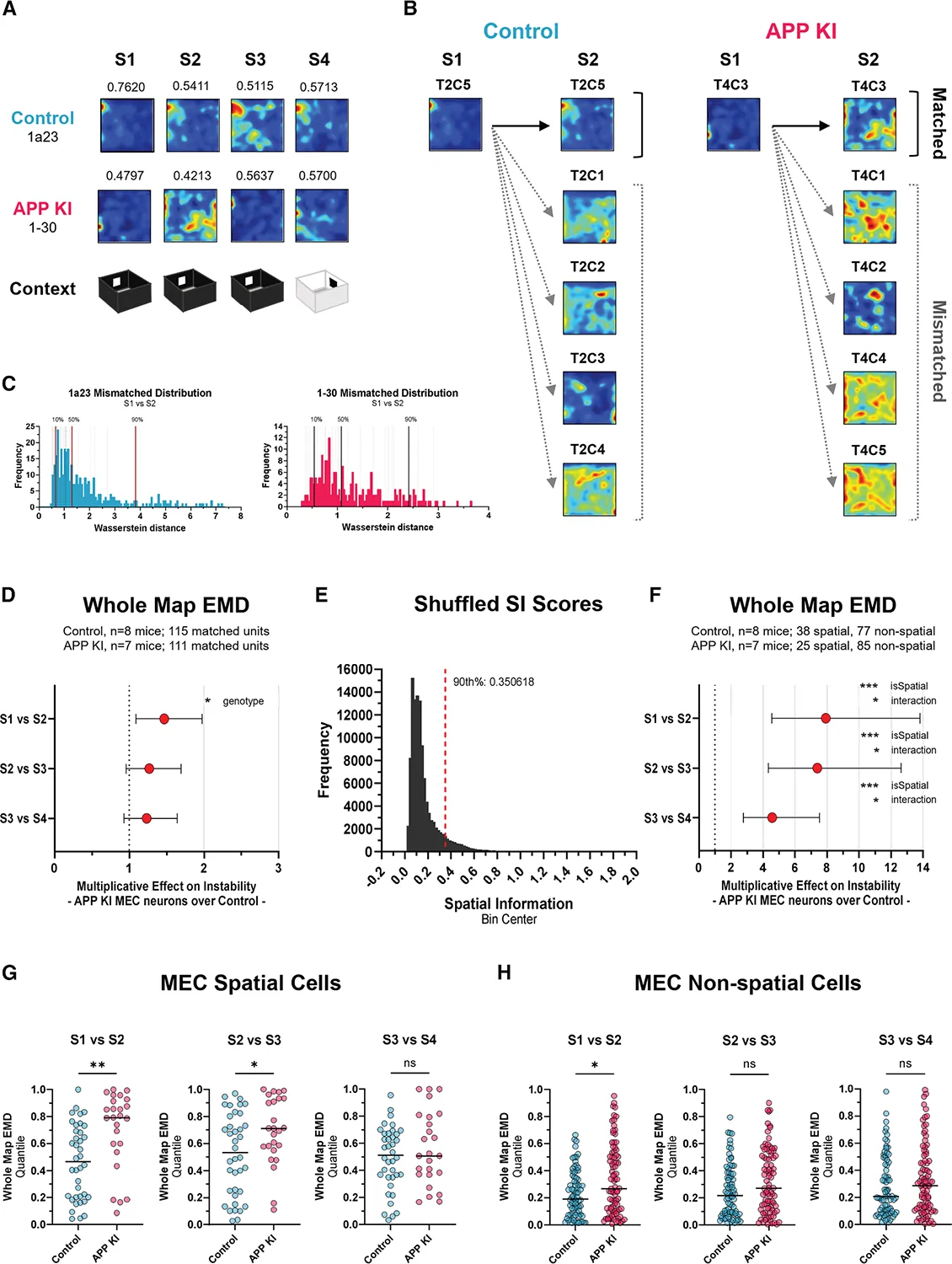
MEC spatial cells in aged APP KI mice exhibit increased representational instability in familiar environments (A) Ratemaps across all four sessions for two example units. Top, control; bottom, APP KI. SI scores above each map. (B and C) Example null EMD distributions for one control (B) and one APP KI (C) unit on the S1 vs. S2 comparison. Hashed gray line, true EMD for the matched unit; histogram, false EMDs from mismatched units; red solid lines, 10^th^/50^th^/90^th^ percentiles. Smaller quantile = more stable. (D) Odds ratio for the effect of genotype on whole-map EMD quantile (mixed-effects beta regression; see STAR Methods and Table 1). Effect of genotype on S1 vs. S2 (OR = 1.467, *p* = 0.011), no difference on S2 vs. S3 (OR = 1.269, *p* > 0.05) or S3 vs. S4 (OR = 1.234, *p* > 0.05). (E) Distribution of circularly shuffled SI scores from session 1 matched units (see STAR Methods). Units with true SI above the 90^th^ percentile (0.3506) were classified as spatial cells (control: *n* = 38 spatial, *n* = 77 non-spatial; APP KI: *n* = 25 spatial, *n* = 86 non-spatial). (F) Mixed-effects model with added isSpatial term (Table 1). Significant genotype × isSpatial interactions for every session comparison (S1 vs. S2, OR = 7.92, *p* < 0.05; S2 vs. S3, OR = 7.40, *p* < 0.05; S3 vs. S4, OR = 4.58, *p* < 0.05). isSpatial main effect significant in all comparisons (all *p* < 0.001); genotype main effect not significant (all *p* > 0.05). (G) Whole-map EMD quantiles for spatial cells, compared between genotypes. APP KI > control on S1 vs. S2 (*U* = 245.5, *p* < 0.01) and S2 vs. S3 (*U* = 305, *p* < 0.05); n.s. on S3 vs. S4 (*U* = 407). (H) Same for non-spatial cells: APP KI > control on S1 vs. S2 (*U* = 2,588, *p* < 0.05); n.s. on S2 vs. S3 (*U* = 2,792) and S3 vs. S4 (*U* = 2,987). Two-tailed Mann-Whitney *U* tests in (G)–(H). EMD, Earth Mover’s Distance; SI, spatial information; OR, odds ratio. Red datapoints in OR plots, full-range CIs. Scatterplots, median with full data range. *p < 0.05; **p < 0.01; ***p < 0.001; ns, not significant.

We then asked whether representational stability could be quantified across all matched MEC single units, beyond conventional spatial cell types. To do so, we calculated ratemap differences across key session comparisons using a noise-resistant, rate-robust metric based on the Earth Mover’s Distance (EMD) (Figure 3).^78^ The EMD reflects the minimal cost required to transform one probability distribution into another, where cost is proportional to the amount of “earth” moved and the distance it travels.^79^ Here, ratemap firing densities represent “earth,” and the distance moved between compared ratemaps reflects the change in that density, using a fixed arena dimension (32 × 32) across animals and contexts (Figure 3A).

Because raw EMD scores can vary with individual occupancy differences and spiking variability, we standardized scores by converting them into quantiles for each animal using reference distributions of EMD scores from mismatched units across session comparisons (Figures 3B and 3C; see STAR Methods and Aoun et al. 2024). A mixed-effects beta regression model applied to whole-map EMD quantiles revealed increased instability in APP KI MEC neurons versus controls on first re-exposure to context A (S1 vs. S2: odds ratio = 1.47, *p* < 0.05) but not on the second re-exposure (S2 vs. S3: odds ratio = 1.27, *p* > 0.05) or on novel context exposure (S3 vs. S4: odds ratio = 1.23, *p* > 0.05) (Figure 3D; Table 1).

When matched units were further classified as “spatial” versus “non-spatial” using a circular shuffling method (control: *n* = 38 spatial, 33.04%; APP KI: *n* = 25 spatial, 22.52%) (Figure 3E), significant interaction effects emerged for all session comparisons (S1 vs. S2: *p* < 0.05; S2 vs. S3: *p* < 0.05; S3 vs. S4: *p* < 0.05) (Figure 3F; Table 2). APP KI MEC spatial units were approximately 7.5x more likely than control spatial units to exhibit representational instability in a familiar environment (S1 vs. S2: odds ratio = 7.92; S2 vs. S3: odds ratio = 7.40), and approximately 4.5x more likely to exhibit instability during novel context exposure (S3 vs. S4: odds ratio = 4.60). Whole-map EMD quantile values were significantly larger in APP KI spatial cells versus controls in S1 vs. S2 (*p* < 0.01) and S2 vs. S3 (*p* < 0.05), but not S3 vs. S4 (*p* > 0.05) (Figure 3G). Among non-spatial cells (control: *n* = 77, 66.96%; APP KI: *n* = 86, 77.48%), significant genotype differences were found in S1 vs. S2 (*p* < 0.05), but not in S2 vs. S3 or S3 vs. S4 (*p* > 0.05) (Figure 3H).

We also applied an alternative field-shuffling method to identify spatial units from a distribution of field-shuffled SI scores (Figure S8A).^80^ Significant interaction effects were again found for all session comparisons (S1 vs. S2: *p* < 0.05; S2 vs. S3: *p* < 0.05; S3 vs. S4: *p* < 0.05). Using this method, APP KI spatial units were approximately 8–9 × more likely than controls to exhibit instability in familiar environments (S1 vs. S2: odds ratio = 9.17; S2 vs. S3: odds ratio = 7.78) (control: *n* = 38, 33.04%; APP KI: *n* = 23, 20.72%), and approximately 5x more likely in novel environments (S3 vs. S4: Odds Ratio = 5.18) (Figure S8B; Table S1). Quantile values were significantly larger in APP KI spatial cells in S1 vs. S2 (*p* < 0.001) and S2 vs. S3 (*p* < 0.05), but not S3 vs. S4 (*p* > 0.05) (Figure S8C). Non-spatial APP KI cells also showed significantly larger quantile values in S1 vs. S2 (*p* < 0.05), but not in subsequent comparisons (control: *n* = 77, 66.96%; APP KI: *n* = 88, 79.28%) (Figure S8D).

In our recording paradigm, advanced Aβ pathology impacted the spatial tuning properties of MEC spatial cells differently. Grid cells were predictably impaired, while border and HD cells showed strong tuning properties in all session recordings. However, border cells showed increased instability in both familiar and novel environments despite strong spatial tuning. When quantifying the representational stability of MEC spatial units in our dataset using the whole map EMD, we found that the effect of genotype on MEC neuron instability in familiar and novel contexts varied significantly depending on whether cells were classified as spatial versus non-spatial.

### MEC neurons in aged APP KI mice exhibit poor spatial coding for position and speed

Recent work has shown that EC and hippocampal neurons respond to diverse combinations of task-relevant variables, a property termed mixed-selectivity,^81^ theorized to provide the computational capacity needed for complex thought and action.^82–85^ This framework motivated our investigation into how MEC neuronal populations in aged mice collectively encode multiple behavioral features across familiar and novel environments, and whether Aβ pathology disrupts this ability. To address this, we decoded neuronal activity at the population level, where information encoding is determined from activity patterns of large ensembles rather than individual neurons.^86^

We asked whether spatial information could be decoded from MEC population activity in aged APP KI and control mice as they explored familiar and novel environments (control: *n* = 115; APP KI: *n* = 111 matched units). We used ensembles of one-versus-one linear support vector machines (SVMs) to classify MEC neural population vectors into 25 spatial bins (5 × 5), a standard approach for extending linear SVMs to multi-class settings. Spatial error was computed as the mean squared error (MSE) between predicted and imputed bin centers. Population-level speed decoding was also performed using SVM ensembles, with individual speed distributions divided into 4 bins per mouse per session and speed error computed as the absolute difference of bin centers to ground truth.

**Figure 4.**
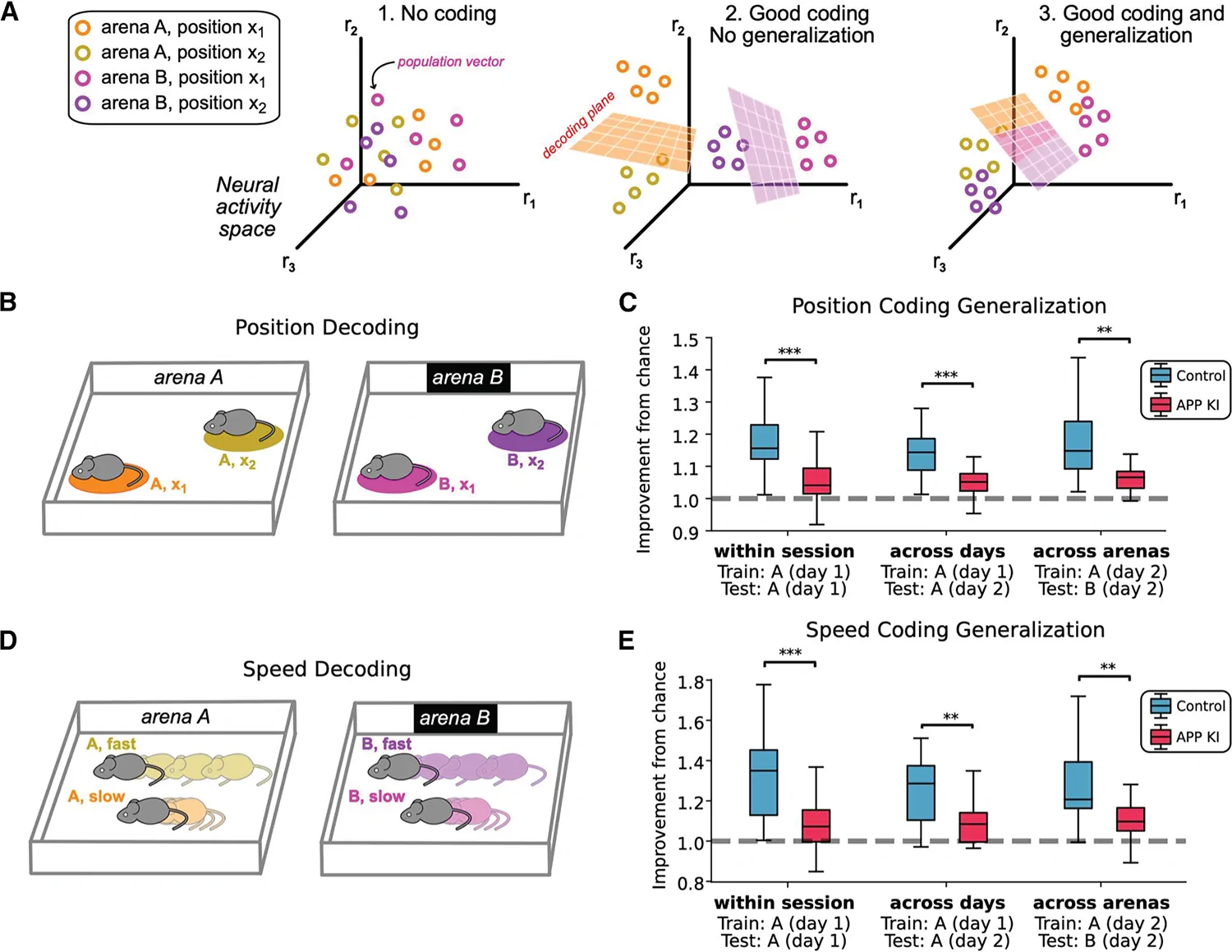
Spatial decoding is impaired across sessions in MEC neurons from aged APP^*NL-G-F/NL-G-F*^ mice (A) Schematic of the three possible decoding outcomes (no coding; coding without across-session generalization; coding with across-session generalization). (B) Schematic: position decoding across two sessions (S3 in context A, S4 in context B). (C) Median position-decoding performance (improvement from chance), control vs. APP KI, by session comparison (S1 vs. S2, *p* < 0.001; S2 vs. S3, *p* < 0.001; S3 vs. S4, *p* < 0.01). (D) Schematic: speed decoding across two sessions. (E) Median speed-decoding performance, by session comparison (S1 vs. S2, *p* < 0.001; S2 vs. S3, *p* < 0.01; S3 vs. S4, *p* < 0.01). Boxplots: interquartile range and median. Wilcoxon signed-rank tests in (C) and (E). **p < 0.01; ***p < 0.001.

Decoding error ratios for APP KI and control MEC neurons were compared using Wilcoxon signed-rank tests. For cross-session generalization of position coding (category 3), significant group differences were found in all session comparisons (S1 vs. S2, *p* < 0.01; S2 vs. S3, *p* < 0.01; S3 vs. S4, *p* < 0.01), with control MEC neurons showing greater improvement from chance than APP KI neurons (Figures 4B and 4C). A similar pattern emerged for speed coding, with control neurons outperforming APP KI neurons on all comparisons (S1 vs. S2, *p* < 0.001; S2 vs. S3, *p* < 0.01; S3 vs. S4, *p* < 0.01) (Figures 4D and 4E). These effects were consistent across all bin conditions tested (16, 25, 36, and 49 spatial bins), with controls outperforming APP KI mice in both position and speed decoding for all session comparisons (Figure S9).

To determine whether poor cross-session generalization in APP KI mice reflected deficits in within-session encoding (categories 1 or 2), we halved each session, trained the decoder on the first half, and tested on the second. Independent runs were performed across 16, 25, 36, and 49 spatial bin conditions. Controls again outperformed APP KI mice in both position and speed decoding across all sessions (25 bins; 4 speed bins) (Figures S10B and S10E). Position decoding error ratios were consistently larger in controls versus APP KI mice across all sessions (S1, *p* < 0.001; S2, *p* < 0.001; S3, *p* < 0.05; S4, *p* < 0.001), as were speed decoding error ratios (S1, *p* < 0.001; S2, *p* < 0.01; S3, *p* < 0.01; S4, *p* < 0.001). Results were consistent using 36 spatial bins and 5–6 speed bins (Figures S10C and S10G), suggesting that reduced cross-session generalization in APP KI mice is driven by overall reduced encoding reliability rather than session-specific inconsistency. Effects were more variable with 16 or 49 spatial bins and 7 speed bins, though overall trends remained consistent with superior decoding performance in Control MEC neurons (Figures S10A, S10D, and S10H). These findings demonstrate that advanced Aβ pathology impairs the ability of MEC neuronal populations to reliably encode and generalize spatial position and speed information across familiar and novel environments, pointing to a fundamental degradation of population-level spatial coding in aged APP KI mice.

### Aged APP KI mice exhibit neuronal hyperactivity in the MEC

Poor spatial coding during exploration of novel and familiar environments in MEC of APP KI mice may be linked to Aβ-associated dysregulation in neuronal firing activity. Indeed, neuronal hyperactivity and network dysfunction have recently been reported in this APP KI mouse model,^56,57,87–91^ building on previous work demonstrating network hyperactivity in transgenic mouse models of Aβ pathology.^54,55,59,61,92^

To examine whether 18-month APP KI mice exhibit single-unit hyperactivity, we calculated firing rates of individual MEC neurons sampled from multiple session 1 recordings across recording blocks and compared them to age-matched controls (see STAR Methods and Data S1). A total of 1,142 MEC neurons were analyzed from 15 mice (control: *n* = 533 units, *n* = 7 mice; APP KI: *n* = 609 units, *n* = 8 mice), with a minimum of 51 neurons sampled per mouse.

**Figure 5.**
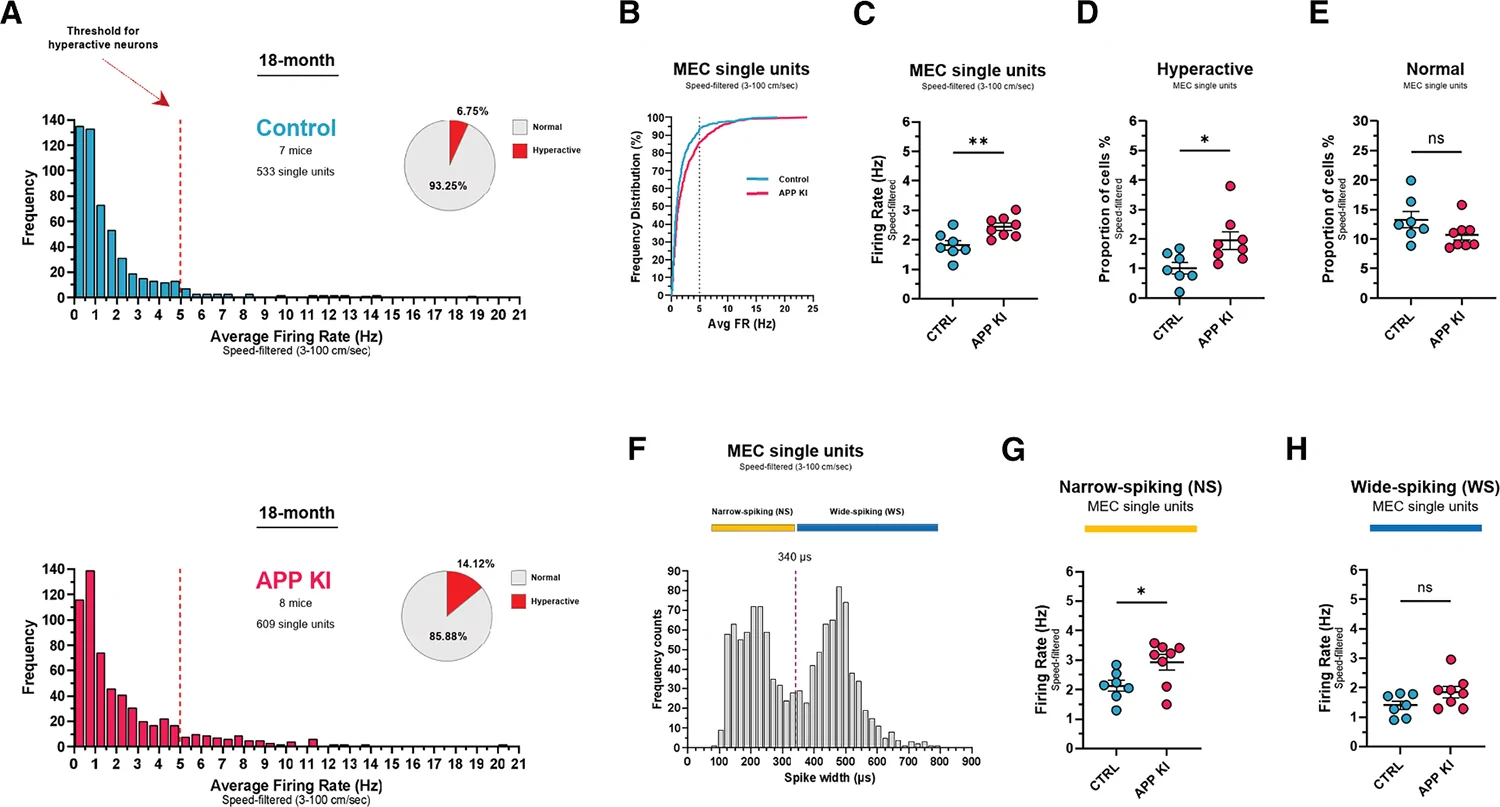
Aged *APP*^*NL-G-F/NL-G-F*^ mice exhibit subtle single unit hyperactivity in the medial entorhinal cortex (A) Frequency distributions of average firing rates (control, top; APP KI, bottom). 0.5 Hz bins. Dashed line: 5 Hz hyperactivity threshold. 14.12% of APP KI vs. 6.75% of Control units exceeded threshold. (B) Cumulative distributions. Kolmogorov-Smirnov test: D = 0.1265, *p* < 0.001. (C) Mean firing rate per mouse. Unpaired *t* test: *t* (13) = 3.099, *p* = 0.0085. (D and E) Proportion of hyperactive (>5 Hz) and normal (<5 Hz) units per mouse. Unpaired *t* tests: hyperactive, *t* (13) = 2.527, *p* = 0.0252; normal, *t* (13) = 1.644, *p* = 0.1242. (F) Firing rate as a function of waveform spike width. Dashed line: ~340 μs split between putative narrow-spiking interneurons (NS, yellow) and wide-spiking excitatory neurons (WS, blue). (G and H) Firing rates of NS (G) and WS (H) units, by genotype. NS, Mann-Whitney *U* test: *U* = 10, *z* = −2.025, *p* = 0.0401. WS, Unpaired *t* test: *t* (13) = 1.836, *p* = 0.0893. Scatterplots show full data range, error bars show standard deviation. *p < 0.05; **p < 0.01; ***p < 0.001; ns, not significant.

Frequency histograms of speed-filtered (3–100 cm/s) AVG FRs revealed a larger proportion of hyperactive neurons (>5 Hz) in APP KI mice (14.12%) compared to controls (6.75%) (Figure 5A). When the proportion of hyperactive (>5 Hz) and normal (<5 Hz) neurons was calculated per animal as a contribution to the group’s total sample, APP KI mice contributed a significantly greater proportion of hyperactive MEC neurons than controls (*p* < 0.05), with no group differences in the contribution of normally firing neurons (Figures 5D and 5E). A two-sample Kolmogorov-Smirnov test comparing cumulative frequency distributions of collective firing rates confirmed a significant shift toward increased firing rates in APP KI neurons versus controls (*p* < 0.001) (Figure 5B). This hyperactive phenotype was further supported by significantly elevated mean firing rates in APP KI MEC neurons compared to controls when averaged per mouse (*p* < 0.01) (Figure 5C).

To examine cell-type-specific firing patterns, we plotted cell frequency as a function of averaged spike width (μs), yielding a bimodal distribution that separated narrow-spiking (NS) from wide-spiking (WS) cells (Figure 5F).^61,93^ NS and WS cells likely correspond to putative interneurons and putative excitatory cells, respectively.^93,94^ A total of 575 NS and 567 WS cells were identified (NS: 50.35%; WS: 49.65% of total neurons). APP KI mice exhibited significantly increased firing rates in NS cells compared to controls (*p* < 0.05) (Figure 5G), whereas no group differences were detected in WS cells (Figure 5H), suggesting the hyperactive phenotype is driven primarily by putative interneurons.

Speed-filtered MEC oscillatory activity was also compared across genotypes in this dataset (control: *n* = 7 mice; APP KI: *n* = 8 mice) (Figure S11). Consistent with cross-session LFP findings (Figure S3), no group differences were detected in percentage power of filtered LFP signals across delta (1–3 Hz), theta (4–12 Hz), beta (13–20 Hz), low gamma (35–55 Hz), or high gamma (65–120 Hz) frequency bands (all *p* > 0.05). Together, these results suggest that advanced Aβ pathology is associated with a mild hyperactive phenotype in MEC of aged APP KI mice that appears to be driven by putative interneurons.

## DISCUSSION

The progressive loss of cognitive function in AD is accompanied by a pervasive increase in behavioral and psychological disturbances, including agitation, restlessness, pacing, and wandering.^95,96^ Spatial disorientation accompanied by wandering behavior has been reported in up to 65% of people with AD residing in assisted-living facilities and is a chief concern for caregivers and a key driver of institutionalization.^97,98^ Although wandering itself is a mid- to late-stage feature, navigation and path-integration deficits emerge early in disease progression and in at-risk individuals (aMCI, *APOE-*ε*4* carriers),^19–21,99–104^ motivating preclinical study of the neural mechanisms underlying spatial-coding decline.

Conventional transgenic mouse models of AD rely on overexpression of FAD-linked mutations and do not faithfully mimic physiological expression patterns, contributing to phenotypic variability in pathology onset, regional deposition, and cognitive deficits.^105,106^ *App* knock-in mouse models target humanized Aβ to the endogenous murine *App* locus, preserving physiological expression.^68^ The *APP*^*NL*^, *APP*^*NL-F*^, and *APP*^*NL-G-F*^ each carry distinct mutations and exhibit distinct Aβ profiles.^68,107^ The *APP*^*NL-G-F/NL-G-F*^ knock-in model stabilizes Aβ oligomers and protofibrils, with plaque onset by ~3 months and near-saturation in hippocampus and cortex by ~7 months. At 6–13 months, *APP*^*NL-G-F*^ mice show deficits in context discrimination and spatial learning and memory, as well as impaired remapping in CA1 place cells and MEC grid cells.^23,57^ Here, we extended this work to *APP*^*NL-G-F*^ (APP KI) mice at 18 months, using a two-day recording paradigm to assess MEC spatial-coding quality and stability across familiar and novel contexts (Figures 1A and 1B).

We found that APP KI MEC neurons encoded poor spatial information across all sessions, independent of prior context exposure (Figures 1D–1F), with cross-session SI correlations revealing coding instability that emerged early and persisted later in the block (Figure S2). Thus, MEC spatial coding in APP KI mice neither improved with repeated exposure nor stabilized across familiar contexts. Grid-cell-like representations were essentially absent in aged APP KI mice (Figures 2A and 2B), replicating prior findings in younger APP KI animals (7–13 months).^23^ Head-direction cells were stable across sessions in both groups (Figures 2E and 2F), whereas border cells in APP KI mice exhibited increased instability in firing preference and spatial tuning (Figures 2C and 2D), and this was not specific to any session transition (familiar re-exposures or novel context). However, no group differences in remapping were observed for any cell type (data not shown).

Disturbed grid cell periodicity in Aβ models has been linked to impaired speed coding and self-motion integration, leading to overreliance on geometric boundaries during navigation.^25^ Indeed, boundary encounters correct spatial errors in grid cell firing (cohesive drift) that accumulate with time and distance since the last detected boundary.^108^ In our paradigm, aged APP KI mice explored arena centers more, and boundaries less, than control mice (Figures S5F and S5G), potentially contributing to increased error accumulation in the grid code through reduced opportunity for drift-based correction. Even when boundaries were encountered, the border cells that would normally anchor spatial coding were themselves unstable in APP KI mice (Figure 2D), providing an additional source of uncertainty in judging contextual familiarity and reducing the utility of an allocentric navigation strategy.

Spatial maps in the EC and hippocampus undergo experience-dependent alterations known as “remapping”,^109–111^ which can affect firing rate alone (rate remapping) or both firing rate and field location (global remapping).^112^ While remapping enables flexible encoding across environments and is thought to support episodic memory, stability of those representations within unchanged environments is also important: consistent spatial maps confer predictability across revisits, which may strengthen memory of familiar environments and support efficient navigation.

To quantify representational stability beyond the limitations of correlation-based methods, we used the whole-map EMD,^78^ normalized using mismatched-unit reference distributions to yield bounded quantiles and modeled the resulting quantiles with a mixed-effects beta regression including genotype and spatial-cell identity (Figure 3; see STAR Methods). Genotype and spatial-cell identity interacted strongly: APP KI MEC spatial cells exhibited greater quantile values—and therefore less stable representations—than controls, with the effect amplified across all session comparisons relative to non-spatial cells (Figure 3F) and strongest during re-exposure to a familiar context (context A: S1–S2, S2–S3) but not on exposure to a novel context (context B: S3–S4) (Figure 3G). Thus, MEC spatial cells in APP KI mice were several times more likely than controls to remap across the recording block, with the strongest effects in familiar rather than novel environments. Similar results were obtained when using an alternative shuffling method to identify MEC spatial cells in our mice (Figure S8). Previous work in mouse models of AD pathology has shown that repeated exposure to a once-novel environment does little to improve spatial stability,^29,30,113^ indicating that excess remapping in Aβ and tau-pathology models likely contributes to the spatial learning and memory deficits observed in these models.

A notable finding was that heightened instability in spatially tuned APP KI MEC units was most pronounced during repeated exposures to a familiar environment (context A: S1 vs. S2, S2 vs. S3) but was not observed during novel context exposure (context B: S3 vs. S4) (Figure 3G). This counterintuitive result likely reflects a convergence of factors that obscured a genotype difference at the S3 vs. S4 transition, including ceiling-level EMD values driven by novelty-induced remapping in both groups, potentially insufficient contextual novelty from exchanging only the arena wall panels, and accumulated map instability in APP KI mice that may have partially mimicked the representational reorganization that novelty would ordinarily produce. At the population level, position- and speed coding were stronger in control than APP KI MEC neurons (Figure 4). Control neurons supported stable decoding both within and across sessions, and for familiar and novel contexts (Figures S9 and S10), whereas APP KI neurons decoded poorly across sessions, indicating a lack of coding generalization across both familiar (S2 and S3) and novel (S4) contexts. Interestingly, APP KI neurons also exhibited poor decoding within sessions (Figure S10), suggesting unstable spatial coding even at fine timescales. Given this within-session instability, the failure of cross-session generalization in APP KI mice is unsurprising.

Decoder accuracy was modest in both groups, which we attributed to behavioral variability during free, open-field exploration. Overall behavioral performance did not differ by genotype (Figures S5B–S5D), though APP KI mice explored arena centers more than control mice in S1 and S3 (morning sessions) (Figures S5F and S5G). Thus, while total arena coverage was similar between genotypes, exploratory coverage differed between subjects, as observed from the individual trajectory maps for each recording block (Figure S4). To address this, the decoder pipeline matched coverage and balanced sample counts per spatial bin across sessions. Nonetheless, decoding accuracy could likely be improved using a linear track, where behavioral variability can be more tightly regulated.

A contentious phenotype in first-generation AD mouse models was nonconvulsive epileptiform-like activity in cortex and hippocampus—interictal spikes (IISs) and related discharges thought to reflect disrupted excitatory/inhibitory balance and network hyperexcitability.^58,59,63,92,114^ Although these events resemble those seen in early-stage AD,^44,115–117^ some preclinical reports have attributed them to APP overexpression during postnatal development rather than to Aβ itself.^118,119^ However, second-generation *APP*^*NL-G-F*^ mice lacking APP overexpression also exhibit interictal epileptiform-like discharges (8–12 months) and neuronal hyperactivity and network dysfunction (3–16 months).^56,57,66,87–91^ Recent *ex vivo* studies in *APP*^*NL-G-F*^ and *APP*^*NL-F*^ brain slices implicate deficits in synaptic signaling and dysregulated homeostatic plasticity as potential drivers of aberrant activity.^60,120,121^ Together, these findings indicate that Aβ-associated disturbances in individual neurons lead to broader circuit and network dysregulation.

Our MEC recordings revealed a mild hyperactive phenotype in aged APP KI mice driven by a subset of NS cells (Figure 5). For this analysis, we pooled >50 MEC neurons per mouse from multiple session 1 recordings (Data S1). Frequency distributions of average firing rates revealed a small population of units that diverged at 5 Hz, with APP KI showing a ~2-fold increase in ≥5 Hz units versus control (14.12% vs. 6.75%) (Figure 5A). These percentages are provided as context for the group-level firing rate distributions. Comparing the cumulative frequency distributions of neuronal AVG FRs per group revealed a shift toward increased firing rates in APP KI neurons versus control (Figure 5B). These findings were supported by further analysis of individual mice, which serve as the primary inferential analyses for group comparisons, with APP KI mice showing increased firing rates and a greater proportion of hyperactive neurons than controls (Figures 5C–5E). Segregating MEC units by spike widths revealed increased firing rates in putative inhibitory interneurons (NS units) of APP KI mice, with no effect on putative excitatory cells (WS units) (Figures 5F–5H).

Several factors could underlie the elevated firing rates of MEC NS units in aged APP KI mice. One possibility is decreased inhibition of MEC interneurons via disruptions in GABAergic signaling from the medial septum. Gonzalez-Sulser et al. (2014) have shown that the medial septum sends monosynaptic inhibitory projections primarily to MEC interneurons and AD models show functional and anatomical remodeling of medial septum-hippocampal GABAergic projections.^122–125^ Thus, Aβ associated septocortical GABAergic transmission could disinhibit MEC interneurons, producing the modest firing-rate increase observed here. A second possibility considers the unique susceptibilities of inhibitory interneuron subtypes to AD pathology. somatostatin-positive (SST) and parvalbumin-positive (PV) interneurons show stage-specific vulnerabilities that may be region and layer-specific.^60,65,126–132^ SST interneurons are selectively vulnerable early in AD,^126,130,133^ whereas PV interneurons remain relatively unaffected until the later stages.^60,130,134^ Although extracellular recordings cannot resolve subtypes, the NS hyperactivity we observe may reflect this differential susceptibility under advanced Aβ pathology. Finally, the NS hyperactivity may represent a late-stage compensatory response to hyperexcitability in neighboring principal neurons. Aberrant excitatory activity likely peaked before 18 months, driving remodeling of inhibitory circuits and increased firing rates in local interneurons.^57,59^ Despite extensive Aβ deposition, the NS hyperactivity we observed was mild—possibly a decline from an earlier, more pathological hyperactive state involving excitatory neurons. We speculate that this residual interneuron hyperactivity may still play a significant role in disrupting spatial coding in the APP KI mouse line.

Whether the observed neuronal hyperactivity and spatial instability are drivers of amyloid pathology or consequences thereof, the relationship is likely bidirectional and synergistic. Accumulating Aβ pathology can disrupt synaptic homeostasis and precipitate an excitatory/inhibitory (E/I) imbalance. This resulting network hyperactivity can, in turn, accelerate the release and deposition of Aβ, creating a deleterious feedback loop. Regardless of the initiating mechanism, whether septocortical circuit disruption, differential interneuron subtype vulnerability, or compensatory remodeling, this sustained elevation in interneuron activity likely degrades network dynamics by reducing the signal-to-noise ratio required for precise spatial coding, thereby perpetuating instability of the cognitive map and underpinning the spatial coding deficits we observed. Our findings underscore the importance of further research into interneuron dysfunction and its contribution to information coding deficits in AD.

### Limitations of the study

First, this study used a single age group (18-month). Younger cohorts would be useful in establishing the onset and progression of spatial coding deficits in this mouse line. Initial project planning included the investigation of MEC spatial coding in two age groups: a young (3–4 months) and an aged (16–18 months) group. We focused on the advanced age group because these results build on previous findings of Aβ-associated impairment in spatial cell remapping, entorhinal-hippocampal circuit dysfunction, and spatial learning and memory in younger APP KI mice.^23,57,88^ Thus, we would expect that EMD and spatial decoding analyses would also yield deficits in younger APP KI cohorts. In fact, the mild elevation in NS unit firing rates at 18 months is consistent with the interpretation that hyperactivity in this model likely peaks at earlier disease stages and may have partially plateaued by advanced age, as excitatory drive diminishes and inhibitory circuits undergo compensatory remodeling.^57,59^ Indeed, prior work in younger *APP*^*NL-G-F*^ mice (3–16 months) has documented more prominent network hyperexcitability and interictal epileptiform-like discharges during the period of active plaque accumulation,^56,57,66,87–91^ suggesting that the spatial coding deficits we report here may be compounded by more severe earlier hyperactivity.

Second, remapping might have been more effectively triggered by altering the arena shape in the fourth recording session of the block. We used uniform 50 × 50 cm square arenas throughout the four-session blocks to keep consistent dimensions for the EMD and decoding analyses across sessions. Contextual novelty was provided by wall panels with unique high-contrast visual cues (Figure S1), allowing new combinations to be used for each recording block per mouse. However, we cannot rule out that the mice experienced some familiarity with nominally “novel” contexts, or conversely that they failed to develop full familiarity with Context A after repeated exposures (S1–S3). Mice may also have attended to non-wall cues (e.g., dim lighting, white-noise localization, and odor), providing stable spatial references that suppressed remapping.

Finally, it is important to distinguish between the observation of network-level instability and the presence of overt cognitive decline. The disrupted representations provide a plausible neural substrate for spatial disorientation but do not unequivocally prove concurrent behavioral failure. Instead, these deficits may represent a prodromal MEC vulnerability. That is, a degradation of spatial-map reliability that precedes or precipitates cognitive impairment, rendering navigation susceptible to failure under high cognitive demand.

In conclusion, advanced Aβ pathology interferes with MEC spatial information coding during exploration of familiar and novel environments. Individual MEC neurons showed reduced spatial information and increased instability, population-level position and speed coding were impaired, and a subset of putative inhibitory interneurons exhibited elevated firing rates. An important next step will be continued use of knock-in lines that preserve native expression of disease-associated mutations.^68,135–137^ These lines are especially useful for probing pathology-associated circuit dysfunction and cognitive impairment.

## RESOURCE AVAILABILITY

### Lead contact

Further information and requests for resources and reagents should be directed to and will be fulfilled by the lead contact, S. Abid Hussaini (syh7006@med.cornell.edu).

### Materials availability

This study did not generate new unique reagents or mouse models.

### Data and code availability

- Source data for all figures will be made freely available on the Hussaini Lab GitHub page (https://doi.org/10.5281/zenodo.19040105).
- All original code used for data analysis will be made freely available on the Hussaini Lab GitHub page.
- Any additional information regarding data is available from the lead contact upon request.

## STAR★METHODS

### EXPERIMENTAL MODEL AND STUDY PARTICIPANT DETAILS

The homozygous *App*^*NL-G-F/NL-G-F*^ knock-in (APP KI) mice used in these studies feature a humanized murine Aβ sequence containing three familial AD mutations: the Swedish (KM670/671NL), Beyreuther/Iberian (I716F), and Arctic (E693G) mutations, and have been described previously.^68^ Original homozygous *App*^*NL-G-F/NL-G-F*^ males and females were obtained via material transfer agreement (MTA) with Drs. Takashi Saito and Takaomi C. Saido (RIKEN Center for Brain Science, Japan). Homozygous mice were backcrossed to C57BL/6J mice (JAX Strain #: 000664) every 10 generations to reduce genetic drift. F10 or greater heterozygous *App*^*NL-G-F/wt*^ breeding pairs were then crossed to regenerate homozygous *App*^*NL-G-F/NL-G-F*^ mice, and the colony was refreshed in this way every 10 generations. All homozygous APP KI mice used in these studies were sampled randomly from available aged cohorts in our colony and genotyped prior to the experiments described. Age-matched C57BL/6J mice served as controls for these studies and were randomly sampled from a colony maintained by the Hussaini lab. To maintain this line, breeding pairs were swapped out after 5 litters and new breeders were purchased from the Jackson Laboratory every 6-months to minimize genetic drift within the control C57BL/6J colony. A total of 18 mice, including males and females, were used. Total mouse numbers per genotype were as follows: *App*^*NL-G-F/NL-G-F*^ mice (Total, n=10; Female, n=6; Male, n=4) and age-matched, non-transgenic C57BL/6J mice (Total, n=8; Female, n=5; Male, n=3).

All mice were housed in a temperature and humidity-controlled vivarium at Columbia University Irving Medical Center and maintained on a standard 12 hr light/dark cycle with food and water provided *ad libitum*. All animal experiments were performed during the light phase in accordance with national guidelines (National Institutes of Health) and approved by the Institutional Animal Care and Use Committee of Columbia University (IACUC Approval Number: AABF3553. The Animal Welfare Assurance number: A3007-01).

### METHOD DETAILS

#### Microdrive construction and tetrode implantation

Microdrives were constructed as described previously.^26,61^ Briefly, custom-made reusable 16-channel microdrives (Axona, UK) were outfitted with tetrodes consisting of twisted, 25-μm-thick platinum-iridium wires (California Fine Wire, USA). Prior to surgery, the tetrodes were cut to an appropriate length and electroplated with Platinum Black plating solution (Neuralynx, USA) until individual channel impedances dropped within a range of 150–200 Kohms. Final impedances were recorded in sterile 0.9% saline solution.

For electrode implantation, mice were first weighed and then anesthetized with isoflurane (3%–4% for induction; 0.5%–3% for maintenance) using a multichannel VetFlo Traditional Anesthesia vaporizer (Kent Scientific, USA) and fixed within a stereotaxic frame (Kopf Instruments, Germany). An intradermal injection of bupivacaine (Marcaine, 2mg/kg) was then administered at the clipped surgical site, followed by an incision to expose the skull 5 min later. Jeweler’s screws were inserted into the skull to support the microdrive implant. A 2-mm hole was made on the right side of the skull at position 3.0–3.1 mm lateral to lambda and approximately 0.2 mm in front of the transverse sinus (right hemisphere). An additional screw connected with wire was inserted into the skull (left hemisphere), serving as a ground/reference. The prepared microdrive was then tilted at 6°–7° in the sagittal plane on a stereotaxic arm and the tetrodes lowered into the brain 1.0 mm from the surface (below dura). The microdrive ground wire was then soldered to the skull screw wire and the microdrive was secured with dental cement. Mice were then allowed to recover in a cleaned cage atop a warm heating pad until normal breathing and ambulation had returned before being transported to non-barrier housing for post-operative monitoring. Mice received Carprofen (5 mg/kg) prior to surgery and postoperatively to reduce pain, in addition to a subcutaneous sterile saline injection to aid in hydration. All implanted animals were housed individually to prevent damage to their microdrives and recording experiments began approximately 1 week from the time of surgery.

#### Experimental design: Recording blocks

*In vivo* recording sequences consisted of recording blocks made up of four individual recording sessions spread over two consecutive days, with two recording sessions performed each day (Figure 1B). Sessions 1 and 2 were recorded on Day 1 in the morning and afternoon, respectively. Sessions 3 and 4 were recorded in the morning and afternoon of the following day (Day 2). The inter-session interval per day was ~4–6 hr and the time interval between days (Session 2 to 3) was ~16–18 hr. Once a recording block was completed for a particular recording depth, tetrodes were advanced 50 to 100μm into the MEC to sample neuronal activity from a new population of neurons. All mice were allowed 48 hr before the next recoding block was performed.

As illustrated in Figure 1B, Session 1 is the first exposure to Context A (novel), in this case depicted as a square arena with black walls and a white cue card affixed to the North wall. Sessions 2 and 3 constitute re-exposures to Context A (familiar), while Session 4 is an exposure to a novel panel arrangement (Context B), depicted in Figure 1B as a square arena with white walls and a black cue card affixed to the East wall. The individual panels used to construct the walls (boundaries) of square open field arenas were white and black 3/16” sturdy foam board (50cm × 80cm) cut from larger one-piece boards (Staples, USA). Panels featured high contrast visual cues, though solid white or black panels with no cues were also used. 80cm white composite corner trim (90° angle) was used at each panel end to assemble a square arena. The variety of panels allowed construction of unique arena contexts for repeated *in vivo* recording sessions while maintaining arena dimensions. Arena dimensions once assembled: 50cm × 50cm × 80cm. All panel arrangements used in the experiments described here are found in Figure S1.

The subject order for daily recordings was randomly assigned so that the same sequence of animals was never repeated across days, and every effort was made to balance the subject order by genotype and sex for each recording block. To begin a recording block (Day 1), mice were transported in their home cages to an isolated recording room within the housing facility and allowed to acclimate for ~30 min. Once a square arena was configured (Context A) and a subject order was established for the day, mice were removed from their home cages, connected to the recording system, and allowed to freely explore the open field for 20 min. Motivated foraging behavior and open field coverage were encouraged by scattering chocolate crumbs throughout the arena. Once Session 1 was completed, mice were gently lifted out of the arena and disconnected from the recording system before being placed back into their home cages. Session 2 recordings were performed under the same experimental conditions and followed the same subject order. The following day (Day 2), the subject order was again randomized prior to recording. Session 3 recordings were performed under the same conditions and in the same arena as Day 1 recordings. Once completed, a novel contextual environment (Context B) was created by assembling a new arena with a panel configuration that differed from Context A. Session 4 recordings were then performed in Context B under the same conditions as prior recordings. The arena space was thoroughly cleaned between each recording session to minimize the influence of odors from a previously run animal. All recordings were performed under low light conditions (ambient room, ~25 lux; center of arena, ~8 lux) with background white noise (50db).

#### *In vivo* recording and single unit analysis

Neuronal signals from our mice were recorded using the Axona DacqUSB system and described previously.^26^ Briefly, recording signals were amplified 10,000 to 30,000 times and bandpass filtered between 0.8 and 6.7 kHz. Spike sorting was performed offline using TINT cluster-cutting software and KlustaKwik automated clustering tool. The resulting clusters were then refined manually and were validated using autocorrelation and cross-correlation functions as additional separation tools. Single units with no undershoot in their waveform were discarded.

A total of 1,142 MEC neurons were collected across 15 mice for data shown in Figure 5 (Control, n=533 single units (Total, n=7 mice; Female, n=5 mice; Male, n=2 mice); APP KI, n=609 single units (Total, n=8 mice; Female, n=4 mice; Male, n=4 mice)). This dataset was generated by sampling MEC neuronal activity from multiple Session 1 recordings across different recording blocks for each mouse. This approach allowed us to capture new populations of MEC neurons in each mouse while ensuring consistency in session sampling across animals. First, a minimum–maximum speed filter (3–100 cm/sec) was applied to remove artifacts due to LED jumps and single unit spikes that occurred during bouts of behavioral immobility. Next, quantitative measurements of cluster quality were performed, yielding isolation distance values in Mahalanobis space.^139^ Median isolation distance values were then calculated per mouse followed by comparison across genotype. No significant difference in cluster quality was detected between groups (median isolation distances: Control, 8.390; APP KI, 7.887: Mann-Whitney *U* test: *U* = 20, *P* = 0.3969). Average firing rates were calculated by dividing the total number of speed-filtered spikes by the duration of recording session (1,200 sec). A conservative threshold for single unit hyperactivity (5 Hz) was determined based on a previous finding in 16-month EC-Tau/hAPP mice (4.74 Hz) and hAPP mice (5.60 Hz) after examining the relative change in proportions of neurons in APP KI mice versus Controls.^55,61^ Putative excitatory neurons (WS) were distinguished from putative interneurons (NS) in this dataset by first examining the frequency distribution histogram of pooled MEC single unit spike widths and then bisecting the binned waveform spike widths after the first modal distribution.^61,93,94^ This resulted in a delineation cutoff at 340μs (Figure 5F). A total of 575 NS cells and 567 WS cells were identified from our MEC recordings (NS, 50.35% versus WS, 49.65% of total neurons). For a detailed breakdown of the electrophysiology dataset, please see Data S1.

A total of 352 single units were isolated across these recording blocks (Control, n=183 units, n=8 mice, n=5 female, n=3 male; APP KI, n=169 units, n=7 mice, n=4 female, n=3 male). Of these, 226 MEC neurons across 15 mice were identified and tracked across sessions in recording blocks described in Figure 1 thru 4, Figures S1 thru 10, Table 1, and Table S1: Control, n=115 matched single units; APP KI, n=111 matched single units. ‘Matched’ neurons were any neurons that appeared in each session of the recording block and could be matched based on cluster profiles in 2D feature space and average waveform. ‘Non-matched’ neurons were any neurons that did not appear in all four sessions of the recording block or those that could not be reliably matched. A total of 126 MEC neurons were classified as Non-matched (Control, n=68 non-matched single units; APP KI, n=58 non-matched single units) and were not included in recording block analysis of tracked neurons.

#### Animal performance

Behavioral analysis of animal performance in the open field arenas has been described previously.^61^ Animal performance during *in vivo* recording sessions was assessed by tracking the position of an infrared LED on the head stage (position sampling frequency, 50 Hz) by means of an overhead video camera. The position data were first centered around the point (0, 0), and then speed-filtered where noted, with only speeds of 3 cm/second or more included in the analysis. Tracking artifacts were removed by deleting samples greater than 100 cm/second and missing positions were interpolated with total durations less than 1 second, and then smoothing the path using a moving average filter with a window size of 300 milliseconds (15 samples on each side). The total distance traveled in the arena (m), % of arena coverage, and average speed (cm/second) during exploration served as dependent measures of interest. For % of arena coverage, the processed position data was plotted and converted to black and white. Using the “bwarea” function in MATLAB, we calculated the total area traveled by the mouse using the binary image and then divided this area by the total area of the arena.

To analyze the amount of time mice spent exploring the center of the maze versus the boundaries, a 4×4 grid was imposed on the arena area and the resulting four centralized boxes were defined as the ‘Center’ zone (625cm^2^). The remaining twelve boxes corresponding to the arena borders were defined as the ‘Boundaries’ zone (1,875cm^2^). Center and Boundaries Zone occupancy (%) was then calculated by summing the time spent in boxes corresponding to each zone as a percentage of the total session duration (20 min). Trajectory maps from each session in the recording blocks are shown for each mouse along with the total distance traveled (m) and % arena coverage (Figure S4). Mouse paths appear yellow on a dark background. A 4×4 grid (white lines) is overlaid onto the trajectory maps. Arena dimensions: 50cm × 50cm.

#### Spatial information and rate map construction

Spatial information content is a measure used to predict the location of an animal from the firing of a neuron. For each neuron, information content was calculated using Skaggs’ formula, which measures the amount of information carried by a single spike about the location of the animal and is expressed as bits per spike.^70^

$$\text{Spatial}\;\text{information}\;\text{content}=\sum Pi\left(\frac{Ri}{R}\right)\text{log}\;2\left(\frac{Ri}{R}\right)$$

where i is the bin/pixel number, Pi is the probability for occupancy of bin i, Ri is the mean firing rate for bin/pixel i and R is the overall firing mean rate.

Firing ratemaps were computed by first making 64×64 unit 2D spatial histograms for occupancy and spike data. Both histograms were then smoothed using a 16×16 unit Gaussian Kernel with a standard deviation of 3.2 units. The smoothed spike map was then divided unit-wise by the smoothed occupancy map to obtain the final rate map. The Peak Firing Rate was defined as the rate in the bin with the highest rate in the firing rate map.

#### Spatial cell analysis and shuffling procedures

Spatial tuning properties of MEC cells were analyzed in Python using the open-source package *opexebo* provided by the Kavli Institute for Neuroscience in Trondheim, Norway (https://pypi.org/project/opexebo/).^140^ MEC spatial cells (e.g. grid, border, HD, and speed) shown in Figure 2 illustrate spatially-selective firing characteristics for their respective cell types described in published reports.^26,73,77,141,142^

MEC spatial cells (e.g. grid, border, HD, and speed) were identified in recording blocks if the matched unit possessed strong spatial tuning in at least one recording session of the block. For grid cells, a unit’s grid score needed to exceed the 95^th^ percentile (0.467576) of a distribution of shuffled grid scores (Total, n=115,000 shuffled scores) on any one session. Border cells were identified if a unit’s border score exceeded the 95^th^ percentile (0.546772) of a distribution of shuffled border scores (Total, n=111,939) on any one session. A limited number of scores (Total, n=3,061) were discarded from the main distribution due to shuffled offsets that resulted in ratemaps with poorly defined fields and spurious border scores. HD cells were identified if a unit’s HD score exceeded the 99^th^ percentile (0.250150) of a distribution of shuffled HD scores (Total, n=114,471) on any one session. A total of 529 shuffled HD scores were discarded from the main distribution due to spurious scores exceeding >0.99. Speed cells were identified if a unit’s speed score exceeded the 99.5^th^ (0.137213, positive speed) or 0.5^th^ (−0.109428, negative speed) percentiles of the distribution of shuffled speed scores. A total of 469 shuffled speed scores were discarded from the main distribution due to returning NaN values. This analysis produced 34 grid cells (Control, n=22; APP KI, n=12), 31 border cells (Control, n=20; APP KI, n=11), 16 HD cells (Control, n=8; APP KI, n=8), 69 negative speed cells (Control, n=29; APP KI, n=39), and 78 positive speed cells (Control, n=33; APP KI, n=45) in our recording block.

Distributions of shuffled spatial scores for each cell type were performed using a circular shuffling procedure on Control Session 1 recording data (Total, n=115 units; n=8 mice). This was accomplished by misaligning spike times and positions by circularly shifting spike timestamps by a constant between 20 seconds and T-20 seconds, where T represents the duration of the recording sessions. This constant was randomly selected for each cell on each of 1,000 shuffles. Ratemaps were recomputed after circular shuffles and a distribution of shuffled spatial scores (Total, n=115,000 shuffled scores) was created per spatial cell type (Figures S6A, S6E, S6I, and S6M).

For EMD analysis (Figure 3), SI scores were used to label ‘spatial’ and ‘non-spatial’ MEC single units using two independent shuffling procedures: circular shuffling and firing field shuffling. For each procedure, spatial cells were labeled ‘spatial’ if their SI scores exceeded the 90^th^ percentile of a distribution of shuffled SI scores on any recording session. This distribution was also created using Control Session 1 recording data (Total, n=115 units; n=8 mice). A total of 114,038 circularly shuffled SI scores were used to create the distribution in Figure 3E from an original total of 115,000 computed scores. Notably, 962 circularly shuffled SI scores were discarded because they exceeded 2.0 SI (range: 2.0–3.99). 640 shuffled scores from mouse 1–28 (T1C1) were discarded, along with 322 shuffled scores from control mouse 1a23 (T2C10). For field shuffling, the positions of segmented firing fields were randomly shuffled within their respective ratemaps using a method described here.^80^ This shuffling method essentially disrupts the original spatial order of firing fields while preserving as best as possible the internal structure of the fields and firing activity. 1,000 field shuffles were performed per unit and a distribution of field shuffled SI scores was created, yielding a total of 115,000 field shuffled SI scores (Figure S8A). The 90^th^ percentile cutoff for the circularly shuffled SI distribution was 0.350618 and resulted in 63 spatial units total (Control, n=38 spatial units; APP KI, n=25 spatial units). The 90^th^ percentile cutoff for the firing field shuffled SI distribution was 0.3589145 and resulted in 61 spatial units total (Control, n=38 spatial units; APP KI, n=23 spatial units). These shuffles informed the labeling of spatial cells later on in the modeling approach described below.

#### Spatial remapping using the Earth Mover’s distance

The Earth Mover’s Distance (EMD) was used to quantify the spatial dissimilarity between two rate map distributions in Figure 3.^78^ The EMD can be thought of as the minimum cost required to transform one distribution into another, where the cost is proportional to the amount of “earth” moved and the distance it is moved. Here, “earth” represents the firing density and the distance represents the change in that density. To efficiently compute this distance on two-dimensional maps, the EMD was approximated using the sliced EMD.^79^ The sliced EMD is computed by projecting the rate map into many random one-dimension slices, and then computing the EMD on each of these slices using the one-dimensional closed-form solution:

$$EMD\left(a,b\right)=\sum_{x,y\in D}f\left(x,y\right)d\left(x,y\right)$$

The average of the distances across multiple slices (10^3^) converges close to the true two-dimensional EMD.

We then computed an EMD metric (Wasserstein distance) across normalized firing rate distributions (ratemaps). The ‘whole map’ EMD distances bins spike counts across the full arena dimensions and is computed for each cell’s pair of rate maps on a given session to session comparison. To standardize the EMD remapping scores, we converted them into quantiles by using animal and session pair specific counterfactual distributions. We defined each counterfactual distribution to be the EMD values calculated from the mismatched neurons of each animal in a specific session to session comparison (i.e. S1 vs S2, S2 vs S3, S3 vs S4). For each animal, and for a given session to session comparison, we computed the EMD between every possible pair of incorrectly matched neurons from those two sessions. We then compared the EMD values from the correctly matched neurons to the distribution of incorrectly matched neurons, yielding quantiles (Aoun et al. 2024). Concretely, we defined the quantile for a correctly matched neuron as the fraction (observed proportion) of mismatched neuron pairs, in the same animal and session-pair comparison, whose EMD score was larger than the correctly matched neuron’s EMD. Thus, lower quantiles indicate more stable representations (smaller EMD relative to mismatches). In other words, the quantile summarizes how much smaller a neuron’s EMD is than expected under random (mismatched) pairings within the same animal and session pair. It is important to note that the quantiles for coherent spatial representations that have been remapped can look similar to quantiles for non-spatial cells. Therefore, the quantiles only serve as a standardized measure of representational stability and not as a measure of spatial information.

To compare standardized remapping scores, we used mixed-effects beta regression models for each session transition because quantile values are bounded between 0 and 1 and because multiple neurons were recorded from the same animal. In addition to distinguishing between genotypes, we categorized MEC single units as spatial cells or non-spatial cells using a binary ‘isSpatial’ variable. This categorical variable with a value of 0 (non-spatial) or 1 (spatial) was defined using circular shuffled distributions of SI scores described previously. A cell was labeled ‘spatial’ if its SI scores exceeded the 90^th^ percentile (0.350618) on any given recording session. This yielded 63 spatial units (Control, n=38; APP KI, n=25) and 163 non-spatial units (Control, n=77; APP KI, n=86) for the EMD analyses in Figure 3. Thus, our beta regression included three fixed effects and one random effect. The three fixed effects were: (1) genotype (control served as the reference state), (2) spatial cell status (1 spatial, 0 non-spatial), and (3) the interaction between (1) and (2). We included the animal ID as a random effect to control for correlations from samples within the same animal. We reported our results as odds ratios derived from the sum of the genotype, spatial cell and interaction effects. Our results thus quantified the impact of APP KI genotype on the stability of spatial representations in spatial cells across recording sessions.

#### Spatial decoding using a support vector machine (SVM)

We assessed the relative spatial decoding performance between groups by training an ensemble of SVMs on single unit activity from each group (Control, n=8 mice/n=115 single units; APP KI, n=7 mice/n=111 single units). We assessed relative decoding performance for two target variables: (1) position, and (2) speed. For position, we divided the animal arena into 16, 25, 36, and 49 spatial bins. For speed, we first determined individual minimum-maximum speed distributions per mouse/session, then divided the distributions into 4, 5, 6, and 7 speed bins. Each target variable and number of bins corresponded to a separate ‘run’ of the pipeline.

During a given run, we divided the dataset into single units from the APP KI group and the Control group. For each group, we trained an ensemble of SVMs using a classification strategy called One Versus One (OVO), where one model is trained per bin and then they are all combined in order to yield the final prediction. For a given decoded label, a set of pseudo-population activity vectors was created by randomly re-sampling and concatenating the neural activity of all individual subjects relative to that specific label. This resampling was performed on a population vector level, i.e., keeping together all neurons recorded simultaneously within individual subjects to preserve possible noise correlations in the neural activity. Importantly, for each run of the decoding analysis, training and testing data were separated within each recorded session before creating the pseudo-population. Finally, to account for unbalanced data, this pseudo-population construction was performed by first down-sampling oversampled labels to ensure that all have the same number of data points. When relevant, only labels with at least a minimum number of data points across all subjects were used for the analysis. Specifically, sessions were subsampled to the minimum coverage among sessions for each spatial bin per condition; bins with fewer than 100 samples were excluded, and 100 samples were drawn from each retained bin to balance the dataset.

The prediction error for each bin was computed in centimeters by taking the average of the Euclidean distances from each predicted bin to the center of the true bin for each target bin. Next, for each bin, the mean error was compared to the mean error from the shuffled predictions within the same training run. Then the ratio of the true model error to the shuffled error was computed for each bin. For visualization, this ratio was expressed as the decoding improvement from chance, such that higher values indicate better-than-chance decoding (Figures 4C and E). Finally, the error ratios for all the bins from the Control group were compared to the bins from the APP KI group using the Wilcoxon signed-rank test (Figures 4C and 4E).

#### Brain tissue fixation and slice preparation

At the end of our experiments, all mice were deeply anesthetized with a cocktail of ketamine/xylazine before being transcardially perfused with ice-cold 100mM phosphate-buffered saline (PBS) pH 7.4., followed by 10% formalin (Fisher Scientific, USA). The last recording position for each microdrive-implanted mouse was noted, followed by careful removal of the microdrive. Mouse brains were then harvested and incubated in 10% formalin overnight, followed by incubation in 30% sucrose until the brains sank to the bottom of a 15mL conical tube (all at 4°C). Following cryoprotection, brain hemispheres from microdrive-implanted mice were separated and frozen in O.C.T. mounting medium. The right hemispheres (implanted) were sliced into sagittal brain sections (40μm) using a Leica CM3050 S cryostat and stored in cryoprotectant at −20°C following immunostaining procedures. Left brain hemispheres (non-implanted) were stored at −80°C.

#### Histology: Immunofluorescence and nissl staining

Immunostained sections in Figure 1 were collected from a naïve, age-matched set of *App*^*NL-G-F/NL-G-F*^ and C57BL/6J mice sampled from our colony. Briefly, free-floating horizontal brain sections (40μm) were rinsed in PBS and then permeabilized with 0.3% Triton X-100 (PBS-T). Sections were then blocked in 10% normal goat serum (NGS) in PBS-T for 60 min at room temperature, followed by overnight incubation at 4°C in primary antibodies diluted in 5% NGS in PBS-T. Primary antibodies used were anti-amyloid beta (Aβ) D54D2 (rabbit monoclonal, 1:1,000) (Cell Signaling, USA) and anti-NeuN (guinea pig polyclonal, 1:5,000) (Synaptic Systems, Germany). The next day, sections were rinsed in PBS-T and incubated in secondary antibodies diluted in 5% NGS in PBS-T. Secondary antibodies used were goat anti-rabbit IgG AlexaFluor 488 (1:500) (Life Technologies) and goat anti-guinea pig IgG Alexa Fluor 594 (1:500). Sections were then incubated in a working solution of Hoechst 33342 dye (5μg/mL) (Thermo Scientific, USA) to stain cell nuclei for 3 min at room temperature. Subsequent washes in PBS were followed by mounting the tissue onto Superfrost Plus slides and coverslipping using SlowFade gold anti-fade reagent (Life Technologies, USA). All slides were stored in the dark at 4°C until imaging.

To verify tetrode placement and reconstruct recording positions in our experimental mice (Figure S12), sagittal tissue sections from implanted hemispheres were mounted onto gelatin coated glass slides and stained in a filtered solution of 0.1% Cresyl violet dissolved in distilled water. Slides were then coverslipped using DPX mountant and stored at room temperature until imaging. We acknowledge the difficulty in matching precise recording locations across individual mice, and so we report a range of tetrode depths per group. The range of MEC recording depths for the recording block dataset was 950μm to 1700μm below dura for Controls and 1050μm to 1500μm below dura for APP KI mice, which we confirmed *post hoc* via histology.

#### Imaging

Fluorescence and brightfield microscopy were performed using an Olympus BX53 upright microscope equipped with an Olympus DP72 12.5 megapixel cooled digital camera. Image files were saved locally on a Dell computer running the Olympus cellSens software platform, then transferred to a Hussaini lab Cyberpower PC for offline image processing in Fiji (Image J).^138^

### QUANTIFICATION AND STATISTICAL ANALYSIS

Statistical analyses were performed in GraphPad Prism (version 10.4.0) and R (version 4.4.1). All datasets were tested for normality using the Shapiro-Wilk test. Datasets in which values were not modeled by a normal distribution were subjected to nonparametric statistical analyses. In Figure 1C, an unpaired *t*-test was used to compare the group means of matched MEC single units (%) tracked in our recording block. Correlation matrices were then assembled to visualize relationships between matched MEC single unit’s SI scores, speed-filtered AVG FRs, and speed-filtered Peak FRs in Figures 1D and 1E. The resulting Pearson’s correlation coefficients (r) were color scaled using the ‘magma’ colormap in GraphPad Prism (1.0, yellow; −1.0, black). Unpaired *t*-tests were used to compare the SI group means for each session in Figure 1F. A mixed-effects beta regression model was used to determine variable effects on the whole map EMD in Figures 3D and 3F. Summary statistics are shown in Table 1. Genotype and isSpatial variables, and their interaction, were considered fixed effects while the Subject ID was included as a random effect to control for correlated samples. The mixed-effects beta regression models (beta distribution with a logit link) were fit in R using the glmmTMB package. Diagnostic tests for dispersion, zero-inflation and outliers were computed using the DHARMa package with non-significant results for all tests. Mann-Whitney *U* tests were used to compare the group median quantile values in each session comparison shown in Figure 3E. Wilcoxon signed-rank tests were used to assess genotype differences in decoding error ratios for position and speed in Figures 4C and 4E. A two-sample Kolmogorov-Smirnov test was used to compare the distributions of speed-filtered AVG FRs in Figure 5B. Unpaired *t*-tests were used to compare group means in Figures 5C–5E and 5H.

Welch’s *t*-tests were used to compare group means of Pearson’s correlation coefficients (*r*) for each session comparison in Figure S2. Repeated measures two-way ANOVAs were used to calculate the effects of two independent variables (genotype × session) on the percentage of power values for individual oscillatory frequency bands in Figure S3. A repeated measures two-way ANOVA was used to calculate differences in performance metrics (Total Distance, Arena Coverage, Avg Speed) over time in Figures S5B–S5D. Correlation matrices of animal performance metrics are shown in Figure S5E. The resulting Pearson’s correlation coefficients (r) were color scaled (1.0, white; −0.5, black). Three-way ANOVAs were used to calculate the effects of three independent variables (genotype × session × zone) on the percentage of time (occupancy), distance traveled (distance), and percent arena covered (coverage) in the arena zones across sessions in the recording block in Figures S5F–S5H. Repeated measures two-way ANOVAs were used in Figures S6C, S6D, S6G, S6H, S6K, S6L, S6O, S6P, S6Q, and S6R to detect group differences in average spatial scores per cell type in our recording block. As previously described for Figure 3, a mixed-effects beta regression model was used to determine variable effects on the whole map EMD in Figure S8B. Summary statistics for this analysis appear in Table S1. Mann-Whitney *U* tests were used to compare the group median quantile values in each session comparison shown in Figures S8C and S8D. Wilcoxon signed-rank tests were used to detect genotype differences in Figures S9 and S10. Unpaired *t*-tests were used to compare means in Figure S11. *Post hoc* analyses were performed using Tukey’s multiple comparisons test where noted.

## Supplementary Material

1

2

Supplemental information can be found online at https://doi.org/10.1016/j.celrep.2026.117505.

## Figures and Tables

**Table 1.** Mixed-effects beta regression of EMD quantile: Genotype-only model

Dependent	session comp.	AIC	BIC	Treatment estimate	Treatment *p* value
EMD quantile	S1 vs. S2	−39.9340	−26.2519	0.3838	0.0115
EMD quantile	S2 vs. S3	−31.3390	−17.6568	0.2390	0.1021
EMD quantile	S3 vs. S4	−24.4748	−10.7927	0.2105	0.1460

Treatment (genotype) estimates and associated p values are shown for each session comparison (S1 vs S2, S2 vs S3, S3 vs S4), along with AIC and BIC values. Values correspond to Figure 3D. Fixed effect: Genotype (treatment); Random effect: Animal ID. AIC, Akaike Information Criterion; BIC, Bayesian Information Criterion; EMD, Earth Mover’s Distance.

**Table 2.** Mixed-effects beta regression of EMD quantile: Genotype × spatial-cell-identity model

Dependent variable	Session comp.	AIC	BIC	Treatment estimate	Treatment p value	isSpatial estimate	isSpatial p value	Interaction estimate	Interaction p value
EMD quantile	S1 vs. S2	−103.4474	−82.9242	0.3599	0.1519	1.0466	2.5379 × 10^−7^	0.6628	0.0289
EMD quantile	S2 vs. S3	−100.7438	−80.2205	0.1969	0.3334	1.0605	1.3721 × 10^−7^	0.7438	0.0134
EMD quantile	S3 vs. S4	−61.5005	−40.9773	0.1519	0.3901	0.7209	5.1153 × 10^−4^	0.6486	0.0372

Genotype (treatment), isSpatial, and interaction estimates with associated p values are shown for each session comparison, along with AIC and BIC values. Values correspond to Figure 3F. Fixed effects: Genotype (treatment), isSpatial, Interaction; Random effect: Animal ID. AIC, Akaike Information Criterion; BIC, Bayesian Information Criterion; EMD, Earth Mover’s Distance.

**Table:** KEY RESOURCES TABLE

REAGENT or RESOURCE	SOURCE	IDENTIFIER
Antibodies		
Rabbit monoclonal anti-amyloid beta (Aβ) D54D2 (1:1,000)	Cell Signaling Technology	Cat#8243; RRID: AB_2797642
Guinea pig polyclonal anti-NeuN (1:5,000)	Synaptic Systems	Cat#266 004; RRID: AB_2619988
Goat anti-rabbit IgG Alexa Fluor 488 (1:500)	Life Technologies (Thermo Fisher Scientific)	Cat#A-11008; RRID: AB_143165
Goat anti-guinea pig IgG Alexa Fluor 594 (1:500)	Life Technologies (Thermo Fisher Scientific)	Cat#A-11076; RRID: AB_141930
Chemicals, peptides, and recombinant proteins		
Platinum Black plating solution	Neuralynx	N/A
Hoechst 33342 nuclear stain (5 μg/mL)	Thermo Scientific	Cat#H3570
10% formalin	Fisher Scientific	N/A
SlowFade Gold anti-fade reagent	Life Technologies	Cat#S36936
O.C.T. compound (Tissue-Tek)	Sakura Finetek	Cat#4583
DPX mountant for histology	Sigma-Aldrich	Cat#44581
Deposited data		
Source data (Data S1): all figure data	This paper	https://zenodo.org/records/19040105
Experimental models: Organisms/strains		
Mouse: *App*^*NL-G-F/NL-G-F*^ knock-in (APP KI): homozygous; humanized Aβ with Swedish (KM670/671NL), Iberian (I716F), and Arctic (E693G) mutations	Saito et al.^68^ 2014	N/A; obtained via MTA from RIKEN Center for Brain Science, Japan
Mouse: C57BL/6J (age-matched control; 18 months)	The Jackson Laboratory	JAX: 000664; RRID: IMSR_JAX:000664
Software and algorithms		
MATLAB	MathWorks	https://www.mathworks.com; RRID: SCR_001622
TINT cluster-cutting software	Axona Ltd.	http://www.axona.com
KlustaKwik automated spike clustering	Kadir et al.	https://github.com/klusta-team/klustakwik
Python	Python Software Foundation	https://www.python.org; RRID: SCR_008394
opexebo v0.4.3 (spatial analysis package)	Kavli Institute for Systems Neuroscience, NTNU	https://pypi.org/project/opexebo/
GraphPad Prism v10.4.0	GraphPad Software	https://www.graphpad.com; RRID: SCR_002798
R v4.4.1	R Core Team	https://www.r-project.org; RRID: SCR_001905
glmmTMB (R package)	Brooks et al.	https://github.com/glmmTMB/glmmTMB
DHARMa (R package)	Hartig and Lohse	https://github.com/florianhartig/DHARMa
Fiji (ImageJ)	Schindelin et al.^138^ 2012	https://fiji.sc; RRID: SCR_002285
Olympus cellSens imaging software	Olympus	https://www.olympus-ims.com/en/cellsens/
Custom code (manual spike curation software, spatial maps, EMD, spike-width classification)	This paper	https://zenodo.org/records/19040105
Other		
Axona DacqUSB 16-channel recording system with custom reusable microdrives	Axona Ltd.	http://www.axona.com
Platinum-iridium wire, 25 μm diameter (tetrode material)	California Fine Wire Company	https://calfinewire.com/item/alloys/all-alloys/100167-platinum-10-percent-iridium-wire
VetFlo Traditional Anesthesia vaporizer	Kent Scientific	https://www.kentscientific.com/products/vaporizer-with-vetflo-single-channel-anesthesia-stand/
Stereotaxic frame	Kopf Instruments	https://kopfinstruments.com/product/model-962-dual-ultra-precise-small-animal-stereotaxic-instrument/
Leica CM3050 S cryostat	Leica Biosystems	https://www.leicabiosystems.com/us/histology-equipment/cryostats/leica-cm3050-s/
Olympus BX53 upright fluorescence/brightfield microscope with Olympus DP72 digital camera	Olympus	https://evidentscientific.com/en/products/upright/bx53

## Highlights

- Aged APP KI mice exhibit poor spatial information content in medial entorhinal cortex
- Increased instability of spatial representations in aged APP KI mice
- Aged APP KI mice exhibit deficits in position and speed decoding
- Aβ pathology is associated with mild hyperactivity in putative interneurons
