# Supplementary material for: Impaired spatial coding and neuronal hyperactivity in the medial entorhinal cortex of aged APP knock-in mice
Source: Cell Rep. Author manuscript; Available in PMC 2026 Sep 10. (PMC13560757; doi:10.1016/j.celrep.2026.117505)
Supplement: Supplementary file 1 [file NIHMS2190745-supplement-1.pdf]

Cell Reports, Volume *45*

Supplemental information

Impaired spatial coding and neuronal hyperactivity in the medial entorhinal cortex of aged APP knock-in mice

Gustavo A. Rodriguez, Andrew Aoun, Eva F. Rothenberg, C. Oliver Shetler, Lorenzo Posani, Srujan V. Vajram, Thomas Tedesco, Anurag Sharma, Stefano Fusi, and S. Abid Hussaini

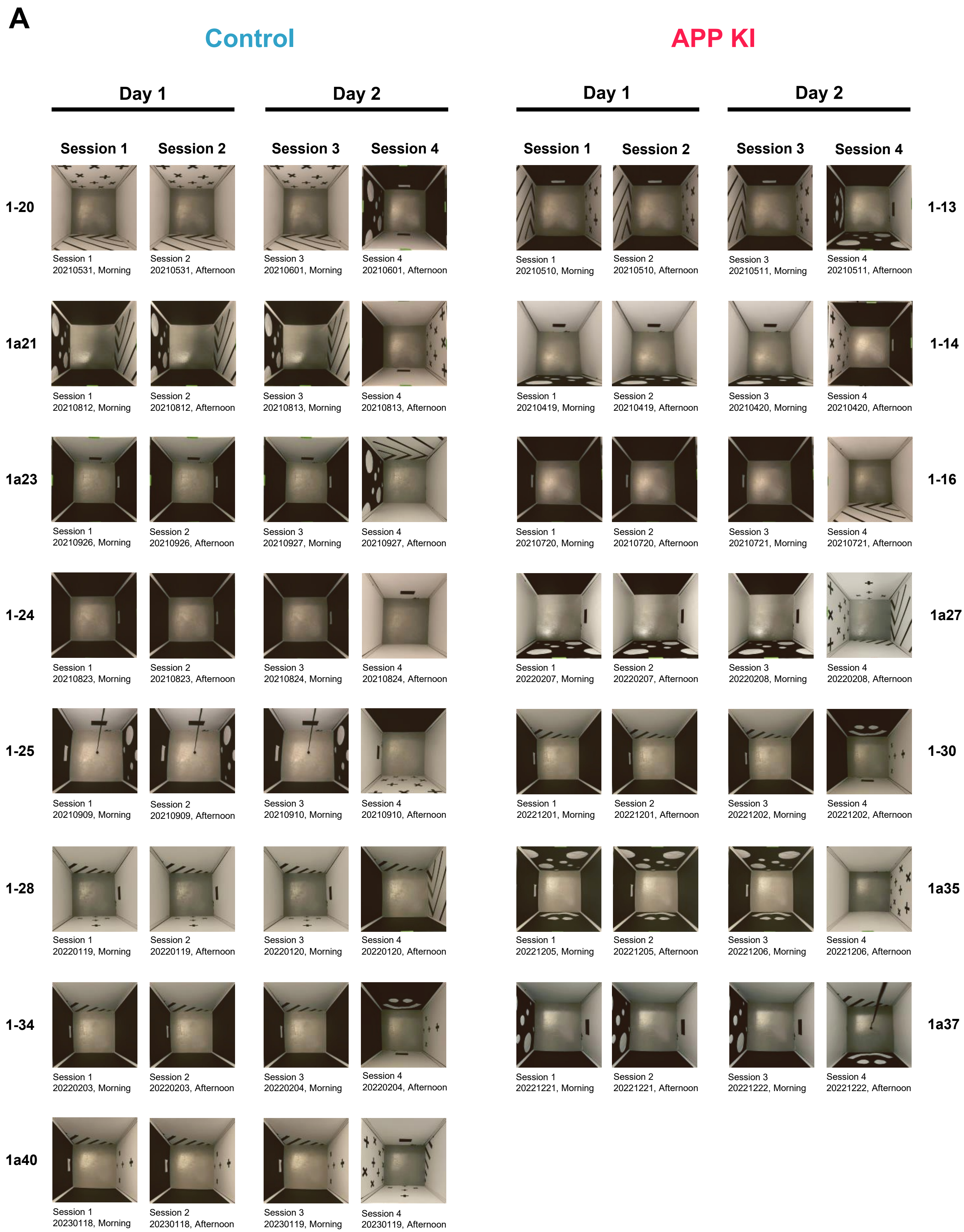
A
Control
APP KI
Day 1
Day 2
Session 1
Session 2
Session 3
Session 4
1-20
1a21
1a23
1-24
1-25
1-28
1-34
1a40
1-13
1-14
1-16
1a27
1-30
1a35
1a37
Session 1 20210531, Morning
Session 2 20210531, Afternoon
Session 3 20210601, Morning
Session 4 20210601, Afternoon
Session 1 20210510, Morning
Session 2 20210510, Afternoon
Session 3 20210511, Morning
Session 4 20210511, Afternoon
Session 1 20210812, Morning
Session 2 20210812, Afternoon
Session 3 20210813, Morning
Session 4 20210813, Afternoon
Session 1 20210419, Morning
Session 2 20210419, Afternoon
Session 3 20210420, Morning
Session 4 20210420, Afternoon
Session 1 20210926, Morning
Session 2 20210926, Afternoon
Session 3 20210927, Morning
Session 4 20210927, Afternoon
Session 1 20210720, Morning
Session 2 20210720, Afternoon
Session 3 20210721, Morning
Session 4 20210721, Afternoon
Session 1 20210823, Morning
Session 2 20210823, Afternoon
Session 3 20210824, Morning
Session 4 20210824, Afternoon
Session 1 20220207, Morning
Session 2 20220207, Afternoon
Session 3 20220208, Morning
Session 4 20220208, Afternoon
Session 1 20210909, Morning
Session 2 20210909, Afternoon
Session 3 20210910, Morning
Session 4 20210910, Afternoon
Session 1 20221201, Morning
Session 2 20221201, Afternoon
Session 3 20221202, Morning
Session 4 20221202, Afternoon
Session 1 20220119, Morning
Session 2 20220119, Afternoon
Session 3 20220120, Morning
Session 4 20220120, Afternoon
Session 1 20221205, Morning
Session 2 20221205, Afternoon
Session 3 20221206, Morning
Session 4 20221206, Afternoon
Session 1 20220203, Morning
Session 2 20220203, Afternoon
Session 3 20220204, Morning
Session 4 20220204, Afternoon
Session 1 20221221, Morning
Session 2 20221221, Afternoon
Session 3 20221222, Morning
Session 4 20221222, Afternoon
Session 1 20230118, Morning
Session 2 20230118, Afternoon
Session 3 20230119, Morning
Session 4 20230119, Afternoon

Supplemental Figure 1. Recording block arena contexts. ***A.*** All panel arrangements that make up arena contexts used in experimental recording blocks are shown along with session date. Individual panels are white and black 3/16" sturdy foam board (50cm x 80cm) that feature various high contrast cues, though solid white or black panels were also used. 80cm white composite corner trim (90° angle) was used at each panel end to assemble a square arena. The variety of panels allowed construction of unique arena contexts for repeated *in vivo* recording sessions while maintaining arena dimensions. Arena dimensions once assembled: 50cm x 50cm x 80cm (LxWxH).

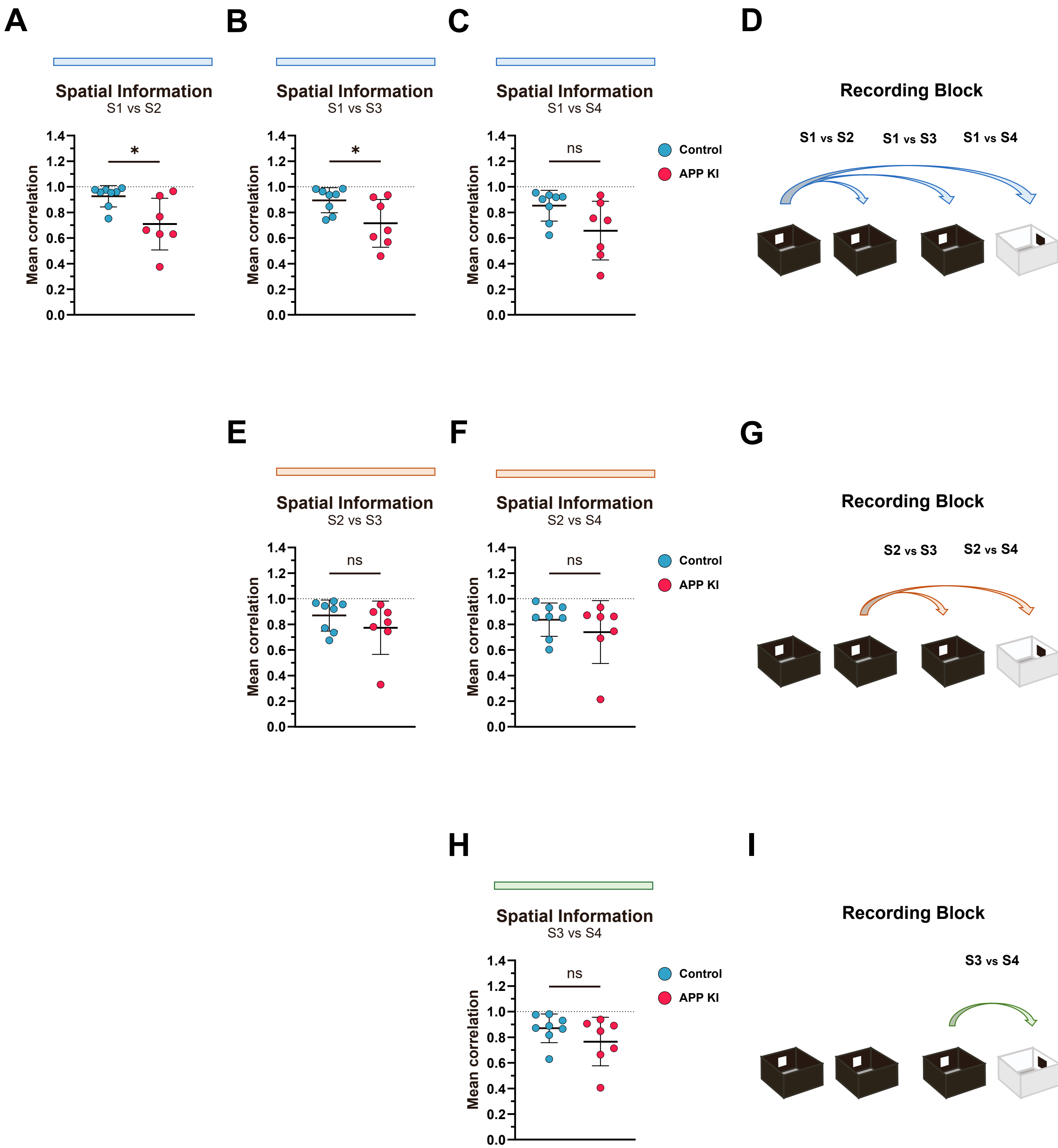

A
B
C
D
Spatial Information
S1 vs S2
Spatial Information
S1 vs S3
Spatial Information
S1 vs S4
Mean correlation
1.4
1.2
1.0
0.8
0.6
0.4
0.2
0.0
*
ns
Control
APP KI
Recording Block
S1 vs S2
S1 vs S3
S1 vs S4
E
F
G
Spatial Information
S2 vs S3
Spatial Information
S2 vs S4
Recording Block
S2 vs S3
S2 vs S4
H
I
Spatial Information
S3 vs S4
Recording Block
S3 vs S4

Supplemental Figure 2. Recording block cross-session correlations of spatial information scores. Spatial information (SI) scores were calculated for each consistently tracked ('Matched') medial entorhinal cortex (MEC) single unit in our recording block. Pearson's correlation coefficient (*r*) was then calculated for all single units per mouse based on session comparisons to assess informational stability across the recording block (Control, n=8 mice; APP KI, n=7 mice). For each session comparison, the group means were compared using Welch's t-test. Overall, cross-session correlations of SI scores from Control MEC single units were quite strong, whereas corresponding correlations of SI scores from APP KI MEC single units were moderate. ***A-C.*** Genotype differences in mean Pearson's *r* values were detected on the following session comparisons: Session 1 vs Session 2 (Control, 0.9267 ± 0.0833; APP KI, 0.7089 ± 0.2016) (t=2.667; $p<0.05$) and Session 1 vs Session 3 (Control, 0.8951 ± 0.0981.; APP KI, 0.7142 ± 0.1866) (t=2.302; $p<0.05$), but not Session 1 vs Session 4 (Control, 0.8521 ± 0.1204; APP KI, 0.6579 ± 0.2289) (t=2.013; $p>0.05$). ***D.*** An illustration of recording session comparisons for A-C. ***E-F.*** No genotype differences in mean Pearson's *r* values were detected in Session 2 vs Session 3 (Control, 0.8684 ± 0.1215; APP KI, 0.7735 ± 0.2086) (t=1.057; $p>0.05$) or Session 2 vs Session 4 (Control, 0.8364 ± 0.1302; APP KI, 0.7393 ± 0.2456) (t=0.937; $p>0.05$) session comparisons. ***G.*** An illustration of recording session comparisons for E-F. ***H.*** No genotype differences in mean Pearson's *r* values were detected in Session 3 vs Session 4 (Control, 0.8701 ± 0.1122; APP KI, 0.7664 ± 0.1893) (t=1.268; $p>0.05$). ***I.*** An illustration of the recording session comparison for H. Scatter plots and numerical values in figure legend represent the mean ± standard deviation of the Pearson's *r* values per mouse in each group. * $p<0.05$.

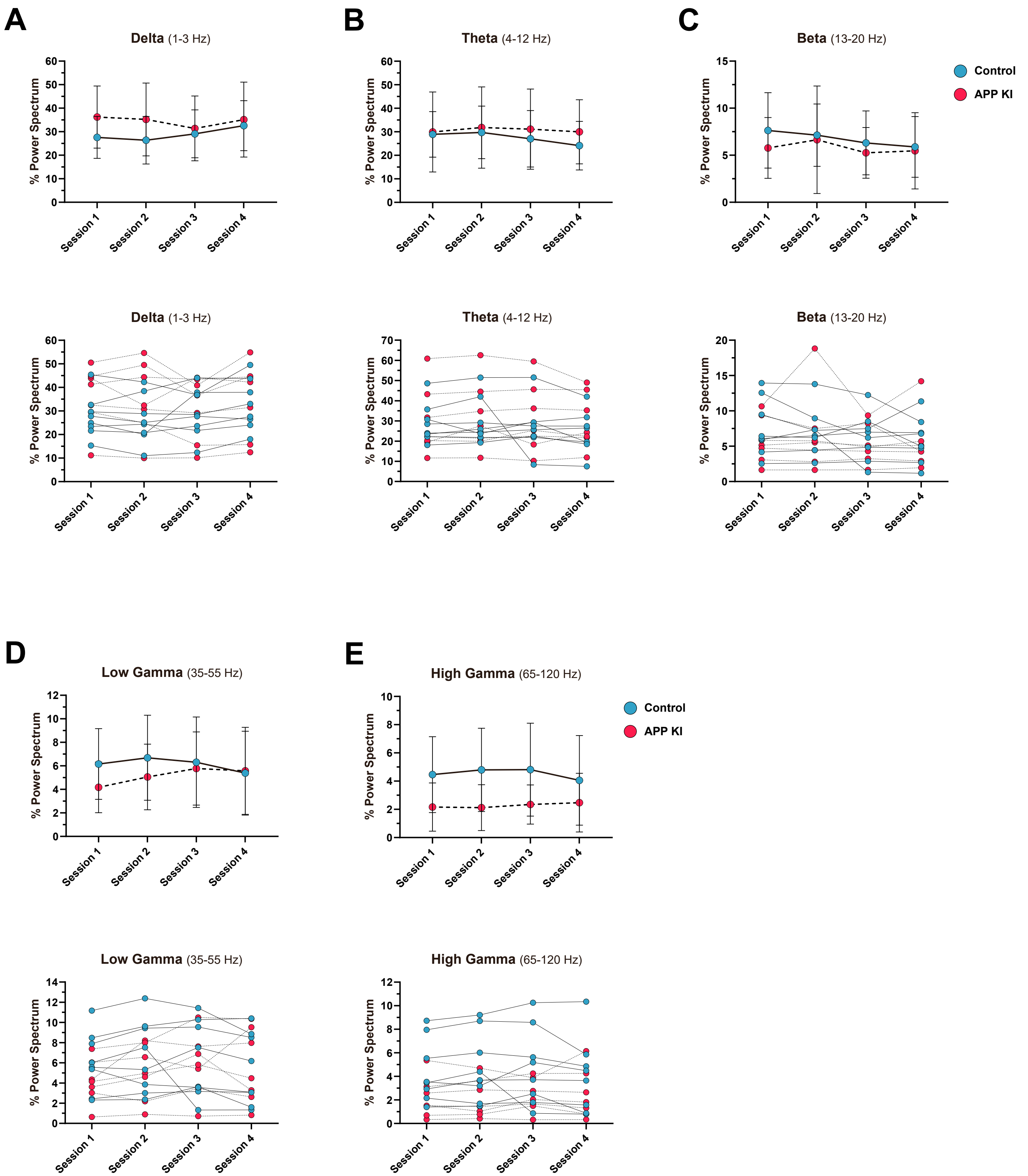

A
Delta (1-3 Hz)
B
Theta (4-12 Hz)
C
Beta (13-20 Hz)
Control
APP KI
% Power Spectrum
Session 1
Session 2
Session 3
Session 4
Delta (1-3 Hz)
Theta (4-12 Hz)
Beta (13-20 Hz)
D
Low Gamma (35-55 Hz)
E
High Gamma (65-120 Hz)
Control
APP KI
Low Gamma (35-55 Hz)
High Gamma (65-120 Hz)

Supplemental Figure 3. No effect of genotype on *in vivo* medial entorhinal cortex oscillatory activity throughout recording block sessions. Local field potentials (LFP) were recorded from the medial entorhinal cortex (MEC) of 18-month *APP* $^{NL\text{-}G\text{-}F/NL\text{-}G\text{-}F}$ (APP KI) mice (n=7) and age-matched C57BL/6J control mice (n=8) as they explored familiar and novel contexts in the recording block. The percentage power values for oscillatory frequency bands were calculated and compared across genotype and recording sessions using a repeated measures Two-Way ANOVA. No significant main or interaction effects were detected at any frequency band. ***A.*** Delta (1-3 Hz): (Session x Genotype) $F_{(3,39)} = 2.735$, $p>0.05$. (Session) $F_{(2.053,26.69)} = 2.093$, $p>0.05$. (Genotype) $F_{(1,13)} = 0.841$, $p>0.05$. ***B.*** Theta (4-12 Hz): (Session x Genotype) $F_{(3,39)} = 0.577$, $p>0.05$. (Session) $F_{(1.408,18.30)} = 1.158$, $p>0.05$. (Genotype) $F_{(1,13)} = 0.224$, $p>0.05$. ***C.*** Beta (13-20 Hz): (Session x Genotype) $F_{(3,39)} = 0.470$, $p>0.05$. (Session) $F_{(2.604,33.85)} = 1.621$, $p>0.05$. (Genotype) $F_{(1,13)} = 0.293$, $p>0.05$. ***D.*** Low Gamma (35-55 Hz): (Session x Genotype) $F_{(3,39)} = 2.091$, $p>0.05$. (Session) $F_{(2.329,30.28)} = 1.300$, $p>0.05$. (Genotype) $F_{(1,13)} = 0.387$, $p>0.05$. ***E.*** High Gamma (65-120 Hz): (Session x Genotype) $F_{(3,39)} = 1.413$, $p>0.05$. (Session) $F_{(1.837,23.89)} = 0.511$, $p>0.05$. (Genotype) $F_{(1,13)} = 3.237$, $p>0.05$. Top line graphs and error bars in each panel represent group means ± standard deviation (SD). Bottom line graphs in each panel depict individual frequency band values for each mouse tracked across sessions. Control, solid connecting lines. APP KI, hashed connecting lines.

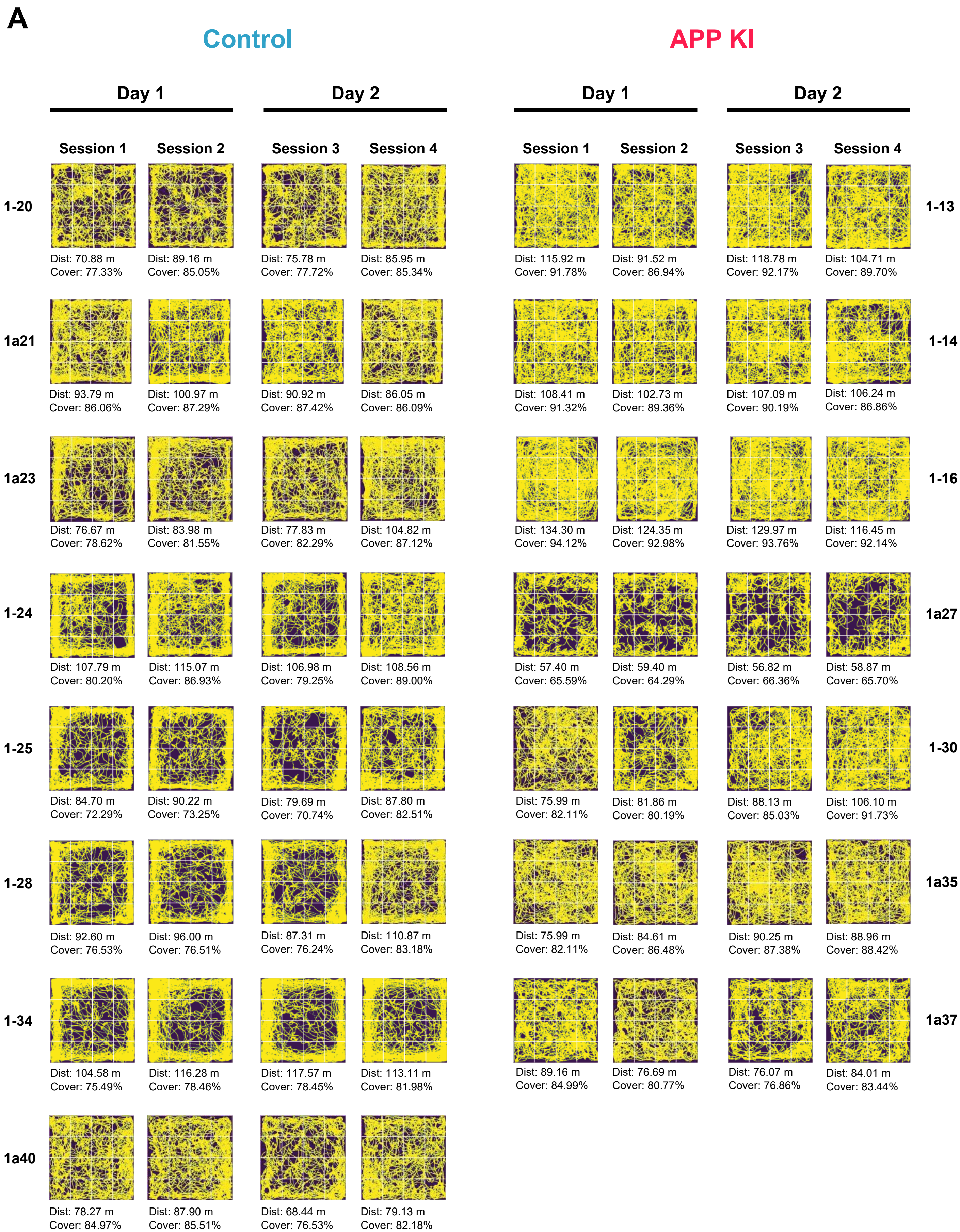
A
Control
APP KI
Day 1
Day 2
Day 1
Day 2
Session 1
Session 2
Session 3
Session 4
Session 1
Session 2
Session 3
Session 4
1-20
Dist: 70.88 m Cover: 77.33%
Dist: 89.16 m Cover: 85.05%
Dist: 75.78 m Cover: 77.72%
Dist: 85.95 m Cover: 85.34%
Dist: 115.92 m Cover: 91.78%
Dist: 91.52 m Cover: 86.94%
Dist: 118.78 m Cover: 92.17%
Dist: 104.71 m Cover: 89.70%
1-13
1a21
Dist: 93.79 m Cover: 86.06%
Dist: 100.97 m Cover: 87.29%
Dist: 90.92 m Cover: 87.42%
Dist: 86.05 m Cover: 86.09%
Dist: 108.41 m Cover: 91.32%
Dist: 102.73 m Cover: 89.36%
Dist: 107.09 m Cover: 90.19%
Dist: 106.24 m Cover: 86.86%
1-14
1a23
Dist: 76.67 m Cover: 78.62%
Dist: 83.98 m Cover: 81.55%
Dist: 77.83 m Cover: 82.29%
Dist: 104.82 m Cover: 87.12%
Dist: 134.30 m Cover: 94.12%
Dist: 124.35 m Cover: 92.98%
Dist: 129.97 m Cover: 93.76%
Dist: 116.45 m Cover: 92.14%
1-16
1-24
Dist: 107.79 m Cover: 80.20%
Dist: 115.07 m Cover: 86.93%
Dist: 106.98 m Cover: 79.25%
Dist: 108.56 m Cover: 89.00%
Dist: 57.40 m Cover: 65.59%
Dist: 59.40 m Cover: 64.29%
Dist: 56.82 m Cover: 66.36%
Dist: 58.87 m Cover: 65.70%
1a27
1-25
Dist: 84.70 m Cover: 72.29%
Dist: 90.22 m Cover: 73.25%
Dist: 79.69 m Cover: 70.74%
Dist: 87.80 m Cover: 82.51%
Dist: 75.99 m Cover: 82.11%
Dist: 81.86 m Cover: 80.19%
Dist: 88.13 m Cover: 85.03%
Dist: 106.10 m Cover: 91.73%
1-30
1-28
Dist: 92.60 m Cover: 76.53%
Dist: 96.00 m Cover: 76.51%
Dist: 87.31 m Cover: 76.24%
Dist: 110.87 m Cover: 83.18%
Dist: 75.99 m Cover: 82.11%
Dist: 84.61 m Cover: 86.48%
Dist: 90.25 m Cover: 87.38%
Dist: 88.96 m Cover: 88.42%
1a35
1-34
Dist: 104.58 m Cover: 75.49%
Dist: 116.28 m Cover: 78.46%
Dist: 117.57 m Cover: 78.45%
Dist: 113.11 m Cover: 81.98%
Dist: 89.16 m Cover: 84.99%
Dist: 76.69 m Cover: 80.77%
Dist: 76.07 m Cover: 76.86%
Dist: 84.01 m Cover: 83.44%
1a37
1a40
Dist: 78.27 m Cover: 84.97%
Dist: 87.90 m Cover: 85.51%
Dist: 68.44 m Cover: 76.53%
Dist: 79.13 m Cover: 82.18%

Supplemental Figure 4. Recording block trajectory maps. ***A.*** Trajectory maps from every recording session in the recording blocks are shown for each mouse along with the total distance traveled (m) and % arena coverage. Mouse paths appear yellow on a dark background. A 4x4 grid (white lines) is overlaid onto the trajectory maps for Center Zone and Boundary Zone occupancy analysis appearing in Supplemental Figure 5. Arena dimensions: 50cm x 50cm.

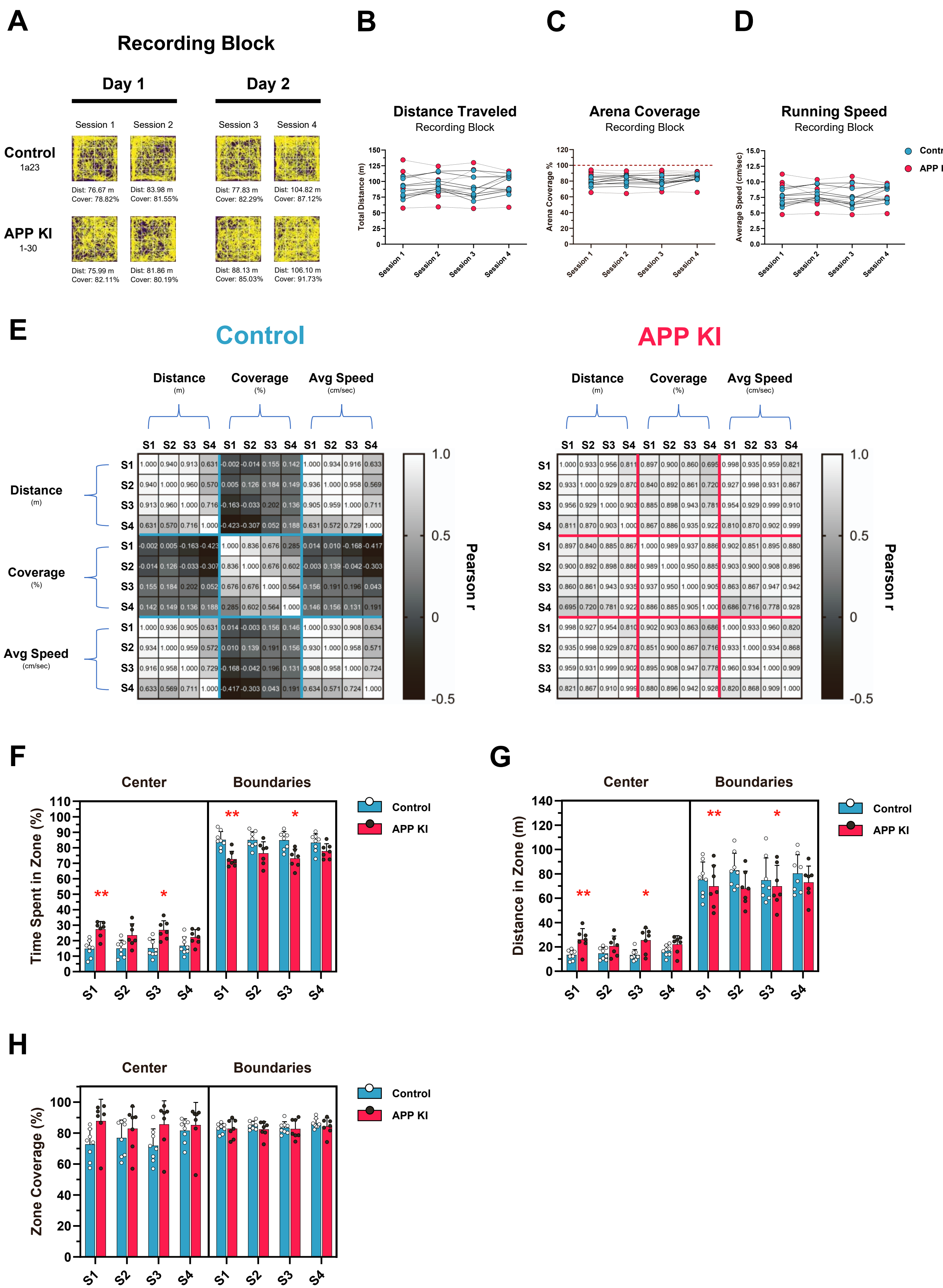
A
Recording Block
Day 1
Day 2
Session 1
Session 2
Session 3
Session 4
Control
1a23
Dist: 76.67 m Cover: 78.82%
Dist: 83.98 m Cover: 81.55%
Dist: 77.83 m Cover: 82.29%
Dist: 104.82 m Cover: 87.12%
APP KI
1-30
Dist: 75.99 m Cover: 82.11%
Dist: 81.86 m Cover: 80.19%
Dist: 88.13 m Cover: 85.03%
Dist: 106.10 m Cover: 91.73%
B
Distance Traveled
Recording Block
Total Distance (m)
C
Arena Coverage
Recording Block
Arena Coverage %
D
Running Speed
Recording Block
Average Speed (cm/sec)
Control
APP KI
E
Control
APP KI
Distance (m)
Coverage (%)
Avg Speed (cm/sec)
S1 S2 S3 S4 S1 S2 S3 S4 S1 S2 S3 S4
Control:
Distance S1 1.000 0.940 0.913 0.631 -0.002 -0.014 0.155 0.142 1.000 0.934 0.916 0.633
S2 0.940 1.000 0.960 0.570 0.005 0.126 0.184 0.149 0.936 1.000 0.958 0.569
S3 0.913 0.960 1.000 0.716 -0.163 -0.033 0.202 0.136 0.905 0.959 1.000 0.711
S4 0.631 0.570 0.716 1.000 -0.423 -0.307 0.052 0.188 0.631 0.572 0.729 1.000
Coverage S1 -0.002 0.005 -0.163 -0.423 1.000 0.836 0.676 0.285 0.014 0.010 -0.168 -0.417
S2 -0.014 0.126 -0.033 -0.307 0.836 1.000 0.676 0.602 -0.003 0.139 -0.042 -0.303
S3 0.155 0.184 0.202 0.052 0.676 0.676 1.000 0.564 0.156 0.191 0.196 0.043
S4 0.142 0.149 0.136 0.188 0.285 0.602 0.564 1.000 0.146 0.156 0.131 0.191
Avg Speed S1 1.000 0.936 0.905 0.631 0.014 -0.003 0.156 0.146 1.000 0.930 0.908 0.634
S2 0.934 1.000 0.959 0.572 0.010 0.139 0.191 0.156 0.930 1.000 0.958 0.571
S3 0.916 0.958 1.000 0.729 -0.168 -0.042 0.196 0.131 0.908 0.958 1.000 0.724
S4 0.633 0.569 0.711 1.000 -0.417 -0.303 0.043 0.191 0.634 0.571 0.724 1.000
APP KI:
S1 1.000 0.933 0.956 0.811 0.897 0.900 0.860 0.695 0.998 0.935 0.959 0.821
S2 0.933 1.000 0.929 0.870 0.840 0.892 0.861 0.720 0.927 0.998 0.931 0.867
S3 0.956 0.929 1.000 0.903 0.885 0.898 0.943 0.781 0.954 0.929 0.999 0.910
S4 0.811 0.870 0.903 1.000 0.867 0.886 0.935 0.922 0.810 0.870 0.902 0.999
S1 0.897 0.840 0.885 0.867 1.000 0.989 0.937 0.886 0.902 0.851 0.895 0.880
S2 0.900 0.892 0.898 0.886 0.989 1.000 0.950 0.885 0.903 0.900 0.908 0.896
S3 0.860 0.861 0.943 0.935 0.937 0.950 1.000 0.905 0.863 0.867 0.947 0.942
S4 0.695 0.720 0.781 0.922 0.886 0.885 0.905 1.000 0.686 0.716 0.778 0.928
S1 0.998 0.927 0.954 0.810 0.902 0.903 0.863 0.686 1.000 0.933 0.960 0.820
S2 0.935 0.998 0.929 0.870 0.851 0.900 0.867 0.716 0.933 1.000 0.934 0.868
S3 0.959 0.931 0.999 0.902 0.895 0.908 0.947 0.778 0.960 0.934 1.000 0.909
S4 0.821 0.867 0.910 0.999 0.880 0.896 0.942 0.928 0.820 0.868 0.909 1.000
Pearson r
F
Center
Boundaries
Time Spent in Zone (%)
G
Distance in Zone (m)
H
Zone Coverage (%)

Supplemental Figure 5. Aged *APP* $^{NL\text{-}G\text{-}F/NL\text{-}G\text{-}F}$ mice exhibit increased exploration in the center zone of open field arenas in recording blocks. Behavioral performance for all 18-month mice through the recording paradigm was first assessed by calculating the total path length (m), percentage of arena coverage (%), and average speed (cm/sec) during exploration for each recording session. ***A.*** Representative trajectories for one mouse per group in the recording block are shown. Control, Mouse ID: 1a23; APP KI, Mouse ID: 1-30. Total distance traveled and % arena coverage values appear below each trajectory map. Trajectory paths appear yellow. ***B-D.*** Behavioral measures were tracked for each mouse in the recording block and analyzed using a repeated measures Two-Way ANOVA. Sample sizes: Control, n=8 mice; APP KI, n=7 mice. No significant differences between genotype were detected on any measure. Repeated measures Two-Way ANOVA: Total distance traveled: (Session x Genotype) $F_{(3,39)}$ = 4.392, p<0.05. (Session) $F_{(3,39)}$ = 1.101, p>0.05. (Genotype) $F_{(1,13)}$ = 0.010, p>0.05. Arena Coverage: (Session x Genotype) $F_{(3,39)}$ = 4.815, p<0.01. (Session) $F_{(3,39)}$ = 4.812, p<0.05. (Genotype) $F_{(1,13)}$ = 1.046, p>0.05. Average speed: (Session x Genotype) $F_{(3,39)}$ = 4.532, p<0.01. (Session) $F_{(3,39)}$ = 1.069, p>0.05. (Genotype) $F_{(1,13)}$ = 0.025, p>0.05. ***E.*** To examine the relationship between total distance traveled, arena coverage, and average speed of our mice in the recording blocks, we generated correlation matrices comparing values for each genotype. All behavioral measures were found to be strongly correlated in APP KI mice across sessions, but not Controls. In Control mice, arena coverage was poorly correlated to distanced traveled and average speed. ***F-H.*** The 50cm x 50cm arenas were divided into 4x4 bins and assigned to one of two zones: Boundaries or Center. All bins along the arena borders were labeled Boundaries (12 bins) and remaining bins in the middle were labeled Center (4 bins). For each mouse and session, the total percentage time, the total distance traveled, and the total coverage of each zone was calculated and analyzed using repeated measures Three-Way ANOVAs. ***F.*** Each group spent significantly greater time exploring the boundaries of the arenas versus the center zone. However, APP KI mice explored the arena center more than Controls. This behavior was also reflected in the percentage time spent along the arena boundaries. Occupancy: (Zone x Session x Genotype) $F_{(3,39)}$ = 2.920, p<0.05. (Zone x Session) $F_{(3,39)}$ = 1.100, p>0.05. (Zone x Genotype) $F_{(1,13)}$ = 15.100, p<0.01. (Session x Genotype) $F_{(3,39)}$ = 0.916, p>0.05. (Zone) $F_{(1,13)}$ = 583.000, p<0.001. (Session) $F_{(3,39)}$ = 0.167, p>0.05. (Genotype) $F_{(1,13)}$ = 5.230, p<0.05. ***G.*** Total distance traveled in the center zone was greater in APP KI mice than Controls. Distance: (Zone x Session x Genotype) $F_{(3,39)}$ = 0.943, p>0.05. (Zone x Session) $F_{(3,39)}$ = 2.280, p>0.05. (Zone x Genotype) $F_{(1,13)}$ = 5.630, p<0.05. (Session x Genotype) $F_{(3,39)}$ = 4.390, p<0.01. (Zone) $F_{(1,13)}$ = 231.000, p<0.001. (Session) $F_{(3,39)}$ = 1.100, p>0.05. (Genotype) $F_{(1,13)}$ = 0.010, p>0.05. ***H.*** No group differences were detected in percent coverage of the center or boundaries zone on any session. Coverage: (Zone x Session x Genotype) $F_{(3,39)}$ = 3.960, p<0.05. (Zone x Session) $F_{(3,39)}$ = 0.804, p>0.05. (Zone x Genotype) $F_{(1,13)}$ = 4.430, p>0.05. (Session x Genotype) $F_{(3,39)}$ = 5.180, p<0.01. (Zone) $F_{(1,13)}$ = 1.500, p>0.05. (Session) $F_{(3,39)}$ = 4.210, p<0.05. (Genotype) $F_{(1,13)}$ = 1.240, p>0.05. Line graphs represent individual mouse performance values across recording block sessions. Bar graphs represent mean ± standard deviation (SD) with individual mouse performance values overlaid on the graph. * p<0.05; ** p<0.01.

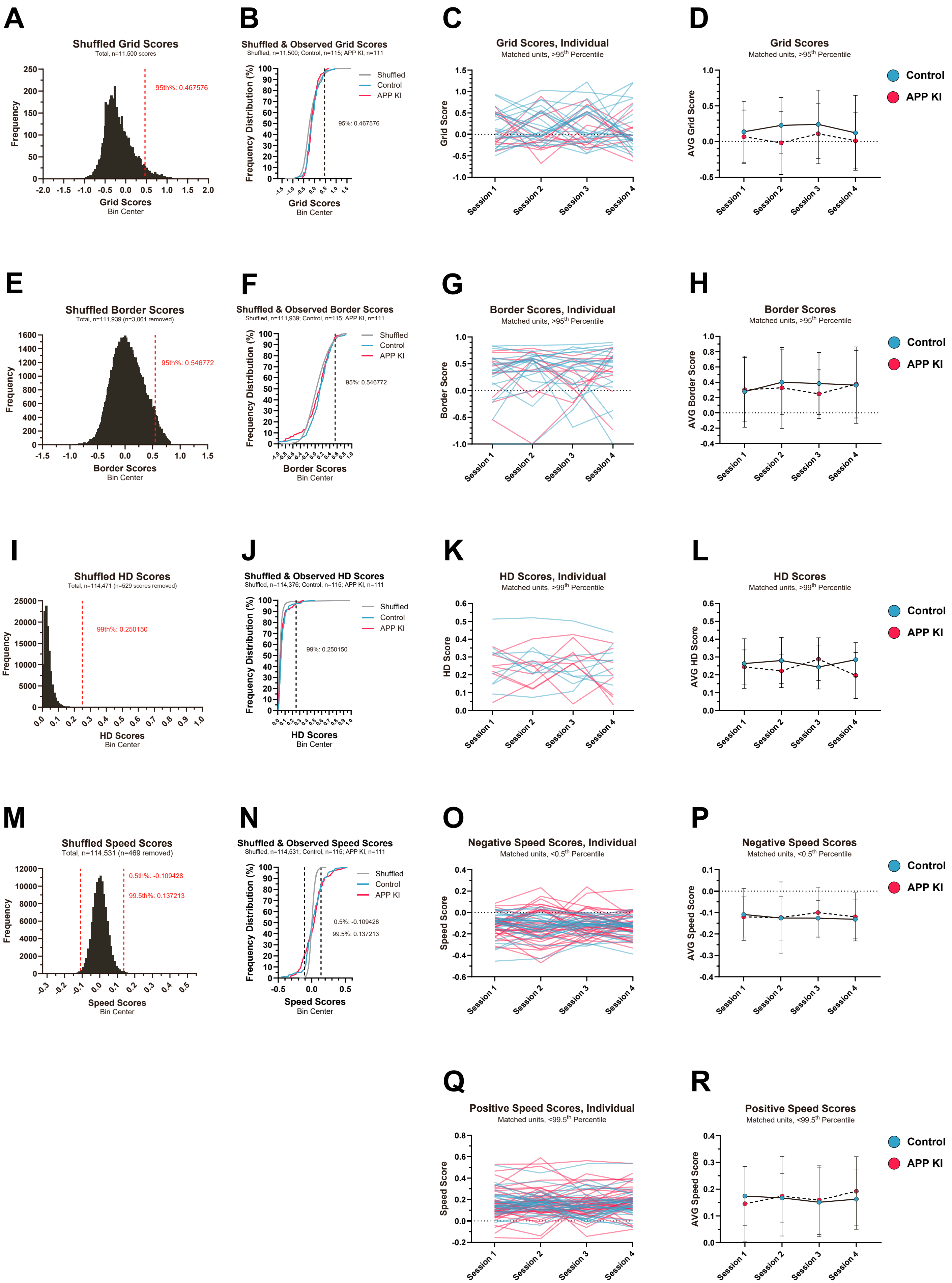

A
Shuffled Grid Scores
Total, n=11,500 scores
95th%: 0.467576
Frequency
Grid Scores
Bin Center
B
Shuffled & Observed Grid Scores
Shuffled, n=11,500; Control, n=115; APP KI, n=111
Frequency Distribution (%)
Shuffled
Control
APP KI
95%: 0.467576
Grid Scores
Bin Center
C
Grid Scores, Individual
Matched units, >95th Percentile
Grid Score
Session 1
Session 2
Session 3
Session 4
D
Grid Scores
Matched units, >95th Percentile
AVG Grid Score
Control
APP KI
E
Shuffled Border Scores
Total, n=111,939 (n=3,061 removed)
95th%: 0.546772
Frequency
Border Scores
Bin Center
F
Shuffled & Observed Border Scores
Shuffled, n=111,939; Control, n=115; APP KI, n=111
95%: 0.546772
G
Border Scores, Individual
Matched units, >95th Percentile
Border Score
H
Border Scores
Matched units, >95th Percentile
AVG Border Score
I
Shuffled HD Scores
Total, n=114,471 (n=529 scores removed)
99th%: 0.250150
HD Scores
J
Shuffled & Observed HD Scores
Shuffled, n=114,376; Control, n=115; APP KI, n=111
99%: 0.250150
K
HD Scores, Individual
Matched units, >99th Percentile
HD Score
L
HD Scores
Matched units, >99th Percentile
AVG HD Score
M
Shuffled Speed Scores
Total, n=114,531 (n=469 removed)
0.5th%: -0.109428
99.5th%: 0.137213
Speed Scores
N
Shuffled & Observed Speed Scores
Shuffled, n=114,531; Control, n=115; APP KI, n=111
0.5%: -0.109428
99.5%: 0.137213
O
Negative Speed Scores, Individual
Matched units, <0.5th Percentile
Speed Score
P
Negative Speed Scores
Matched units, <0.5th Percentile
AVG Speed Score
Q
Positive Speed Scores, Individual
Matched units, <99.5th Percentile
R
Positive Speed Scores
Matched units, <99.5th Percentile

Supplemental Figure 6. Descriptive statistics for MEC spatial cells collected in recording blocks. MEC spatial cells (e.g. grid, border, head-direction, and speed cells) in 18-month mice were identified from distributions of shuffled spatial scores generated by independent runs of shuffling procedures described in the Materials and Methods. Average spatial scores per cell type were then compared across groups using repeated measures two-way ANOVAs. ***A.*** Frequency histogram of shuffled grid scores. The 95th% cutoff score (GS: 0.467576) for grid cell selection is shown as a vertical hashed red line. ***B.*** Cumulative frequency distributions of shuffled and observed grid scores. ***C-D.*** Grid scores for matched MEC units exceeding the 95th% of shuffled scores are shown across sessions in the recording block. Mean grid scores per genotype are also shown. Repeated measures two-way ANOVA: (Session x Genotype) $F_{(2.891,92.51)}$ = 0.2146, $p>0.05$. (Session) $F_{(2.891,92.51)}$ = 0.3093, $p>0.05$. (Genotype) $F_{(1,32)}$ = 3.719, $p>0.05$. Control, n=22 grid cells; APP KI, n=12 grid cells. ***E-H.*** Frequency histogram and cumulative frequency distributions of shuffled and observed border scores are shown with the 95th% cutoff score (BS: 0.546772). Individual border scores for matched MEC units exceeding the 95th% of shuffled scores are shown across sessions, along with group means. Repeated measures two-way ANOVA: (Session x Genotype) $F_{(1.741,50.50)}$ = 0.2873, $p>0.05$. (Session) $F_{(1.741,50.50)}$ = 0.2978, $p>0.05$. (Genotype) $F_{(1,29)}$ = 0.1383, $p>0.05$. Control, n=20 border cells; APP KI, n=11 border cells. ***I-L.*** Frequency histogram and cumulative frequency distributions of shuffled and observed HD scores are shown with the 99th% cutoff score (HD: 0.250150). Individual HD scores for matched MEC units exceeding the 99th% of shuffled scores are shown across sessions, along with group means. Repeated measures two-way ANOVA: (Session x Genotype) $F_{(2.376,16.63)}$ = 1.815, $p>0.05$. (Session) $F_{(2.217,15.52)}$ = 0.2544, $p>0.05$. (Genotype) $F_{(1,7)}$ = 0.5173, $p>0.05$. Control, n=8 HD cells; APP KI, n=8 HD cells. ***M-N.*** Frequency histogram and cumulative frequency distributions of shuffled and observed speed scores are shown, with both 0.5^{th}% cutoff score (-0.109428) and 99.5^{th}% cutoff score (0.137213) shown to capture negative and positive speed cells, respectively. ***O-P.*** Individual speed scores for matched MEC units lower than the 0.5^{th}% of shuffled scores are shown across sessions, along with group means. Repeated measures two-way ANOVA: (Session x Genotype) $F_{(2.575,170.0)}$ = 0.5699, $p>0.05$. (Session) $F_{(2.575,170.0)}$ = 0.3906, $p>0.05$. (Genotype) $F_{(1,66)}$ = 0.1118, $p>0.05$. Control, n=29 negative speed cells; APP KI, n=39 negative speed cells. ***Q-R.*** Individual speed scores for matched MEC units exceeding the 99.5^{th}% of shuffled scores are shown across sessions, along with group means. Repeated measures two-way ANOVA: (Session x Genotype) $F_{(2.600,197.6)}$ = 1.758, $p>0.05$. (Session) $F_{(2.600,197.6)}$ = 1.243, $p>0.05$. (Genotype) $F_{(1,76)}$ = 0.0239, $p>0.05$. Control, n=33 positive speed cells; APP KI, n=45 positive speed cells. For each shuffled distribution, the total number of shuffled spatial scores is reported along with the total number of discarded scores. Line graphs and error bars represent group means ± standard deviation (SD). GS, grid score. HD, head-direction. BS, border score. APP KI, *$APP^{NL\text{-}G\text{-}F/NL\text{-}G\text{-}F}$*. Control, solid connecting lines. APP KI, hashed connecting lines.

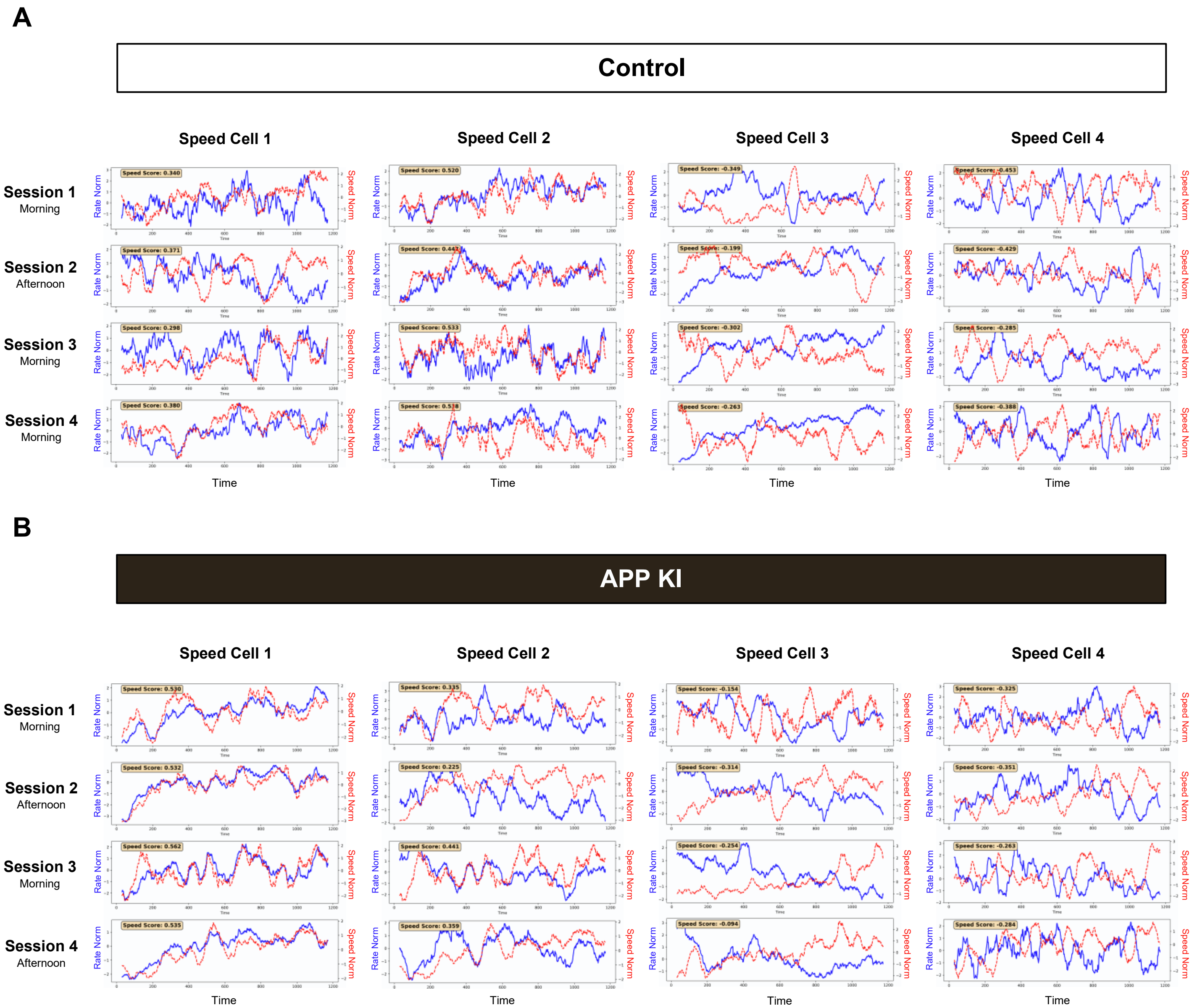

Supplemental Figure 7. Sliding window analysis illustrating normalized firing rate and running speed traces for MEC speed cells. The traces show z-scores for firing rate (blue) and running speed (red) for four representative MEC speed cells per group during free exploration of open field arenas. Activity in full 20 min recording sessions is shown. *Top left*, individual unit/session speed scores are shown. ***A.*** Animal IDs, recording block IDs, and cell IDs are shown for Control: Speed Cell 1, 1-24, S12-13, T3C2. Speed Cell 2, 1-34, S12-13, T3C2. Speed Cell 3, 1-25, S11-12, T3C5. Speed Cell 4, 1-20, S17-18, T3C4. ***B.*** Animal IDs, recording block IDs, and cell IDs are shown for APP KI: Speed Cell 1, 1a27, S16-17, T1C1. Speed Cell 2, 1a27, S16-17, T2C5. Speed Cell 3, 1-13, S17-18, T3C5. Speed Cell 4, 1-14, S7-8, T1C3.

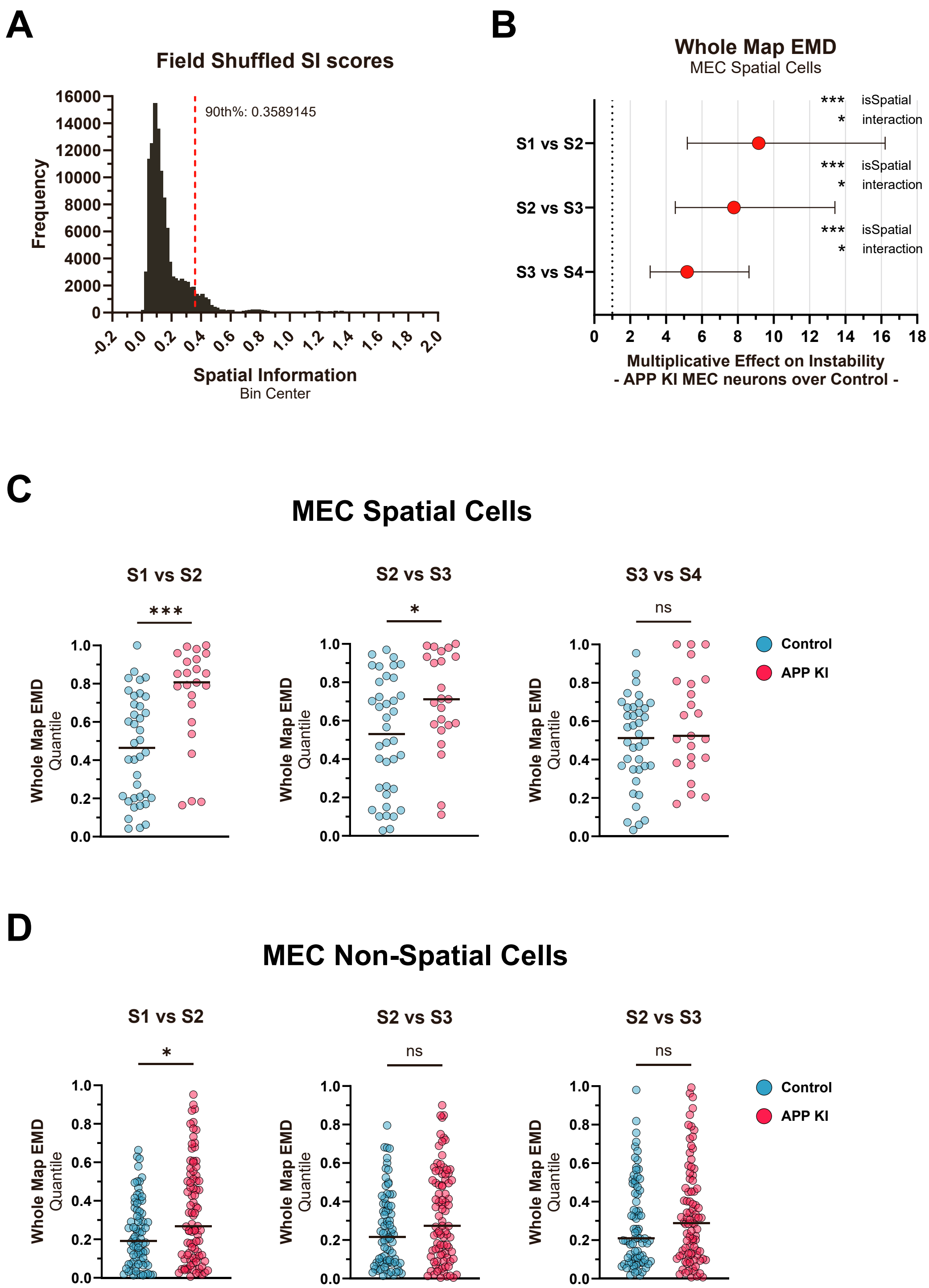
A
Field Shuffled SI scores
90th%: 0.3589145
Frequency
16000
14000
12000
10000
8000
6000
4000
2000
0
-0.2
0.0
0.2
0.4
0.6
0.8
1.0
1.2
1.4
1.6
1.8
2.0
Spatial Information
Bin Center
B
Whole Map EMD
MEC Spatial Cells
*** isSpatial
* interaction
S1 vs S2
*** isSpatial
* interaction
S2 vs S3
*** isSpatial
* interaction
S3 vs S4
0
2
4
6
8
10
12
14
16
18
Multiplicative Effect on Instability
- APP KI MEC neurons over Control -
C
MEC Spatial Cells
S1 vs S2
***
S2 vs S3
*
S3 vs S4
ns
Whole Map EMD
Quantile
1.0
0.8
0.6
0.4
0.2
0.0
Control
APP KI
D
MEC Non-Spatial Cells
S1 vs S2
*
S2 vs S3
ns
S2 vs S3
ns
Whole Map EMD
Quantile
1.0
0.8
0.6
0.4
0.2
0.0
Control
APP KI

Supplemental Figure 8. MEC spatial cells in aged *APP* $^{NL\text{-}G\text{-}F/NL\text{-}G\text{-}F}$ mice identified using an alternative field shuffling procedure exhibit increased representational instability in familiar environments. In Figure 3, medial entorhinal cortex (MEC) spatial cells were identified from a distribution of shuffled spatial information (SI) scores generated by a circular shuffling procedure. This procedure misaligned spike times from *x*,*y* positions by circularly shifting spike timestamps by a constant between 20 seconds and T-20 seconds, where T is the duration of the recording session. As an alternative to this method of circular shuffling, we randomly shuffled the positions of segmented firing fields within their respective ratemaps using a procedure described here (Grieves *et al*., 2021). ***A.*** 1,000 field shuffles were performed for each Control MEC unit in Session 1 of our recording block and a distribution of field shuffled SI scores was created (Total, n=115,000 field shuffled SI scores). All units in Session 1 with true SI scores that exceeded the 90th percentile of this distribution were classified as MEC spatial cells (90th percentile: 0.3589 SI) (Control: n=38 spatial units, n= 77 non-spatial units; APP KI: n=23 spatial units, n=88 non-spatial units). Two APP KI units that were identified as spatial units after circular shuffling did not meet the 90th percentile of field shuffled SI scores and were classified as non-spatial (APP KI: 1-14, T1C2; 1a35, T2C5). ***B.*** Whole map EMD analysis was run as described for Figure 3, with new spatial cell identification after field shuffling. A mixed-effects beta regression model found significant interaction effects of genotype and isSpatial variables for each session comparison (S1 vs S2, OR=9.17, $p<0.05$; S2 vs S3, OR=7.78, $p<0.05$; S3 vs S4, OR=5.18, $p<0.05$). The isSpatial variable had a significant main effect on all session comparisons (S1 vs S2, $p<0.001$; S2 vs S3, $p<0.001$; S3 vs S4, $p<0.001$), while genotype did not (S1 vs S2, $p>0.05$; S2 vs S3, $p>0.05$; S3 vs S4, $p>0.05$). ***C.*** Whole map EMD quantile values for MEC spatial cells identified by field shuffling were compared between genotype for each session comparison using two-tailed Mann-Whitney *U* tests. Quantile values were significantly larger in APP KI MEC spatial cells versus Control spatial cells in S1 vs S2 (U=195.5, $p<0.001$) and S2 vs S3 (U=272, $p<0.05$), but not in S3 vs S4 (U=347, $p>0.05$) comparisons. Sample sizes: Control, n= 38 spatial cells; APP KI, n= 23 spatial cells. ***D.*** Quantile values of MEC non-spatial cells were also compared across genotype. Whole map EMD quantile values were significantly larger in APP KI MEC non-spatial cells versus Control non-spatial cells on S1 vs S2 (U=2,647, $p<0.05$) only. No genotype difference in quantile values were detected on S2 vs S3 (U=2,805, p=0.057) or S3 vs S4 (U=3,058, $p>0.05$) comparisons. Sample sizes: Control, n= 77 non-spatial cells; APP KI, n= 88 non-spatial cells. For EMD analysis, ratemap bins were fixed across all animals and sessions (32X32 bins). EMD, Earth Mover's Distance. OR, Odds Ratio. Red datapoints in EMD OR plots indicate OR values with full range confidence intervals. Scatter plots represent median with full data range. * $p<0.05$; *** $p<0.001$. ns, not significant.

Mixed-effects beta regression model

Fixed effects: Genotype (treatment), isSpatial, Interaction; Random effect: Animal ID

Dependent variable	Session comp.	AIC	BIC	Treatment estimate	Treatment *p* value	isSpatial estimate	isSpatial *p* value	Interaction estimate	Interaction *p* value
EMD quantile	S1 vs S2	-109.1372	-88.6140	0.3681	0.0881	1.0579	1.4981×10^{-7}	0.7896	0.0100
EMD quantile	S2 vs S3	-99.2234	-78.7001	0.2229	0.2639	1.0551	1.7026×10^{-7}	0.7738	0.0118
EMD quantile	S3 vs S4	-65.5817	-45.0585	0.1522	0.3769	0.7226	4.6164×10^{-4}	0.7698	0.0150

Supplemental Table 1. Summary statistics for mixed-effects beta regression models following alternative field shuffling. The table shows the genotype (treatment), isSpatial, and interaction estimates and associated *p* values for each session comparison in Supplemental Figure 6. AIC and BIC values are also shown. Values correspond to Supplemental Figure 6B. AIC, Akaike Information Criteria. BIC, Bayesian Information Criterion. EMD, Earth Mover's Distance.

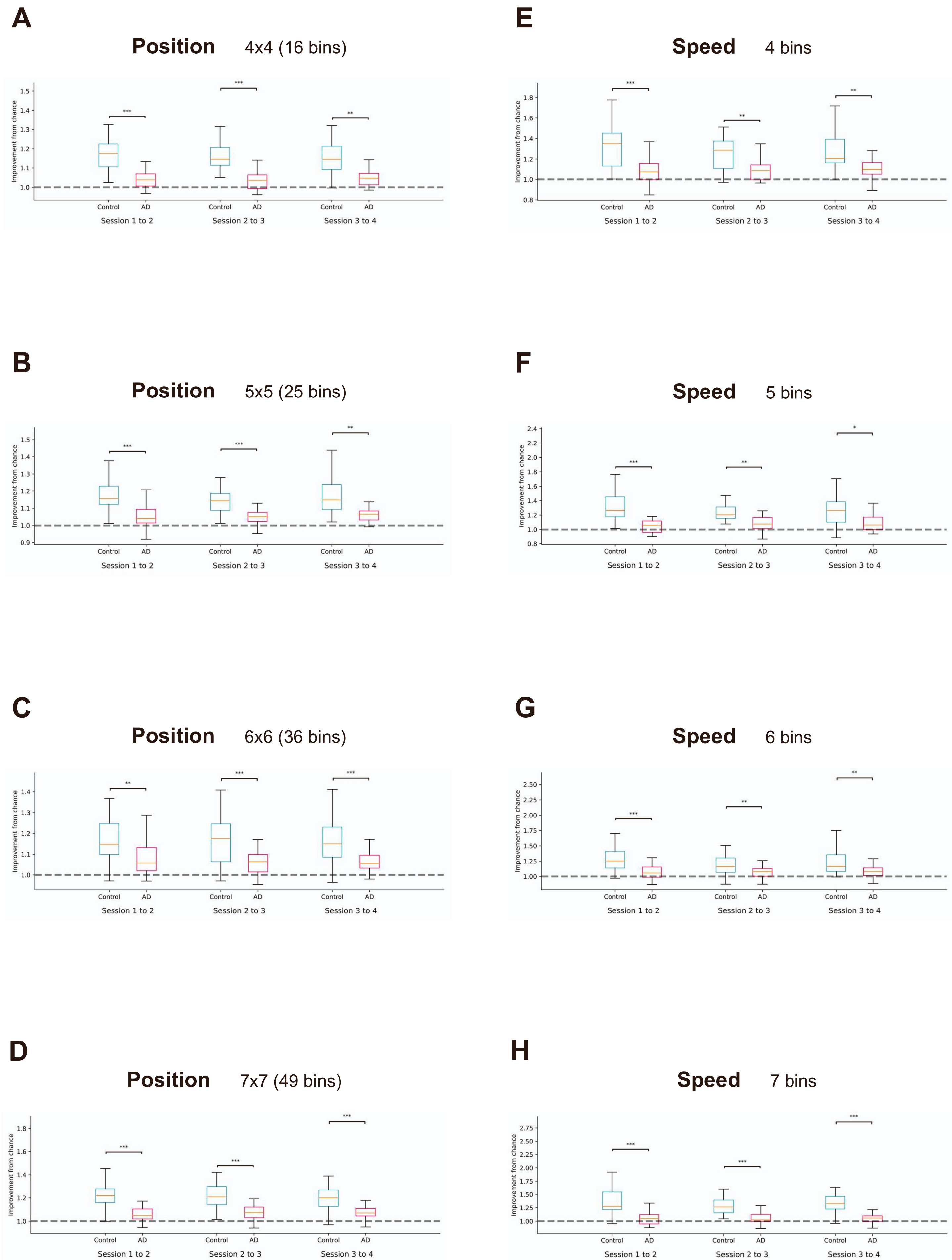
A
Position 4x4 (16 bins)
B
Position 5x5 (25 bins)
C
Position 6x6 (36 bins)
D
Position 7x7 (49 bins)
E
Speed 4 bins
F
Speed 5 bins
G
Speed 6 bins
H
Speed 7 bins
Improvement from chance
Control
AD
Session 1 to 2
Session 2 to 3
Session 3 to 4

Supplemental Figure 9. 18-month *APP* $^{NL\text{-}G\text{-}F/NL\text{-}G\text{-}F}$ mice exhibit impaired stability and generalization of position and speed coding in the medial entorhinal cortex. Linear support vector machines (SVMs) were used to decode discretized position and speed from a pooled set of neurons from aged mice in each group (Control, n=8 mice/n=115 single units; APP KI, n=7 mice/n=111 single units). For cross-session analysis of data collected in our recording block, SVMs were trained on the full first session and tested in the second session for each session comparison (Session 1 vs Session 2, Session 2 vs Session 3, and Session 3 vs Session 4). For position, the arena space (50cm x 50cm) was divided into 16, 25, 36, and 49 spatial bins for individual runs of the analysis. For speed, individual minimum-maximum speed distributions were calculated per mouse/session, then divided into 4, 5, 6 and 7 speed bins for individual runs of the analysis. ***A-D.*** Wilcoxon signed-rank tests were used to detect group differences in median decoding performance for position across sessions, expressed as an improvement from chance level. In all run conditions (spatial bins), MEC neurons from APP KI mice exhibited poor position decoding in each session comparisons versus Control. ***E-H.*** Group differences in median decoding performance for speed were detected in all run conditions (speed bins) and in all session comparisons using Wilcoxon signed-rank tests. MEC neurons from APP KI mice exhibited poor speed decoding for all session comparisons relative to Controls. Total session duration: 20 min. Box plot and whiskers represent the interquartile range (IQR) and median improvement from chance values per genotype for each test half-session comparison. Panels B and E appear in main Figure 4. * $p < 0.05$; ** $p < 0.01$; *** $p < 0.001$.

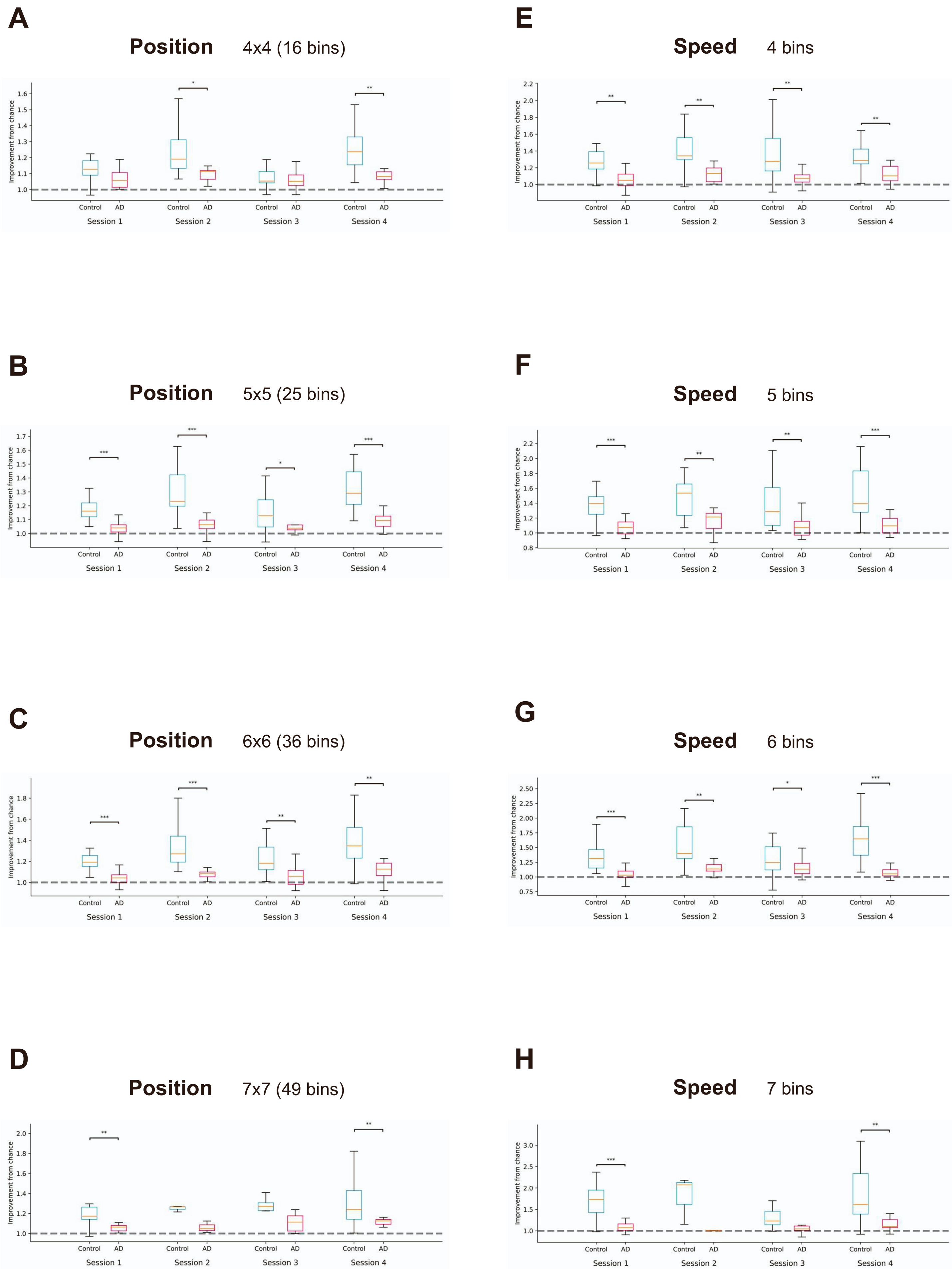

A
Position 4x4 (16 bins)
E
Speed 4 bins
B
Position 5x5 (25 bins)
F
Speed 5 bins
C
Position 6x6 (36 bins)
G
Speed 6 bins
D
Position 7x7 (49 bins)
H
Speed 7 bins
Improvement from chance
Control
AD
Session 1
Session 2
Session 3
Session 4

Supplemental Figure 10. Medial entorhinal cortex neurons from aged *APP* $^{NL\text{-}G\text{-}F/NL\text{-}G\text{-}F}$ mice exhibit impaired spatial coding within individual sessions of the recording block. Linear support vector machines (SVMs) were used to decode discretized position and speed from a pooled set of neurons for 18-month-old animals in each group (Control, n=8 mice/n=115 single units; APP KI, n=7 mice/n=111 single units). For within-session analysis of data collected in our recording block, each recording session was split in half and the SVMs were trained on the first half and assessed on the second half. For position, the arena space (50cm x 50cm) was divided into 16, 25, 36, and 49 spatial bins for individual runs of the analysis. For speed, individual minimum-maximum speed distributions were calculated per mouse/session, then divided into 4, 5, 6 and 7 speed bins for individual runs of the analysis. ***A-D.*** Wilcoxon signed-rank tests were used to detect group differences in median decoding performance for position, expressed as an improvement from chance level, in the tested half of each session. For runs of 25 and 36 spatial bins, MEC neurons from APP KI mice exhibited poor position decoding in each session of the recording block versus Control. ***E-H.*** Group differences in median decoding performance for speed were detected in the tested half of each recording session for runs of 4, 5, and 6 speed bins. For these conditions, MEC neurons from APP KI mice exhibited poor speed decoding performance within each session relative to Controls. Total session duration: 20 min. Half-session duration: 10 min. Box plot and whiskers represent the interquartile range (IQR) and median improvement from chance values per genotype for each test half-session comparison. * $p < 0.05$; ** $p < 0.01$; *** $p < 0.001$.

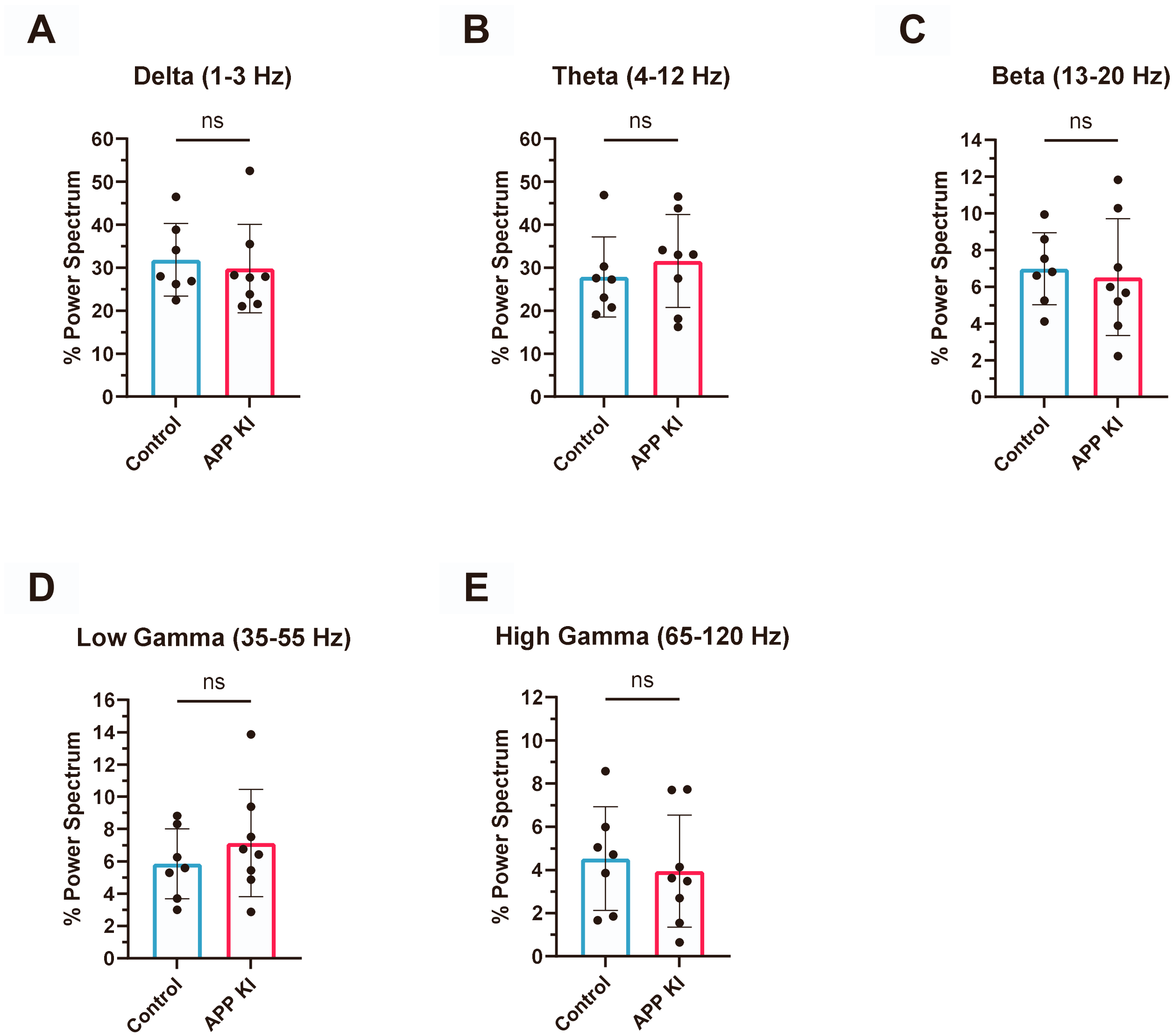

Supplemental Figure 11. No effect of genotype on *in vivo* oscillatory activity in the medial entorhinal cortex of aged experimental mice. Local field potentials (LFP) were sampled in the medial entorhinal cortex (MEC) of 18-month *APP* $^{NL\text{-}G\text{-}F/NL\text{-}G\text{-}F}$ (APP KI) mice (n=8) and age-matched C57BL/6J control mice (n=7). The percentage power values for oscillatory frequency bands were first calculated for individual samples collected from multiple Session 1 recordings across different recording blocks per mouse (see Supplemental Data 1). An average % power spectrum value for each frequency band was then calculated per mouse. Finally, unpaired t-tests were used to compare the averaged % power spectrum values for each frequency band across genotype. No significant group differences were detected at any frequency band. ***A.*** Delta (1-3 Hz): $t_{(13)}$ = 0.4167, P = 0.6837. ***B.*** Theta (4-12 Hz): $t_{(13)}$ = 0.7066, P = 0.4923. ***C.*** Beta (13-20 Hz): $t_{(13)}$ = 0.3337, P = 0.7439. ***D.*** Low Gamma (35-55 Hz): $t_{(13)}$ = 0.8722, P = 0.3989. ***E.*** High Gamma (65-120 Hz): $t_{(13)}$ = 0.4494, P = 0.6606. Bar graphs represent mean ± standard deviation (SD). ns, not significant.

A

C57BL/6J (Control)
18-month

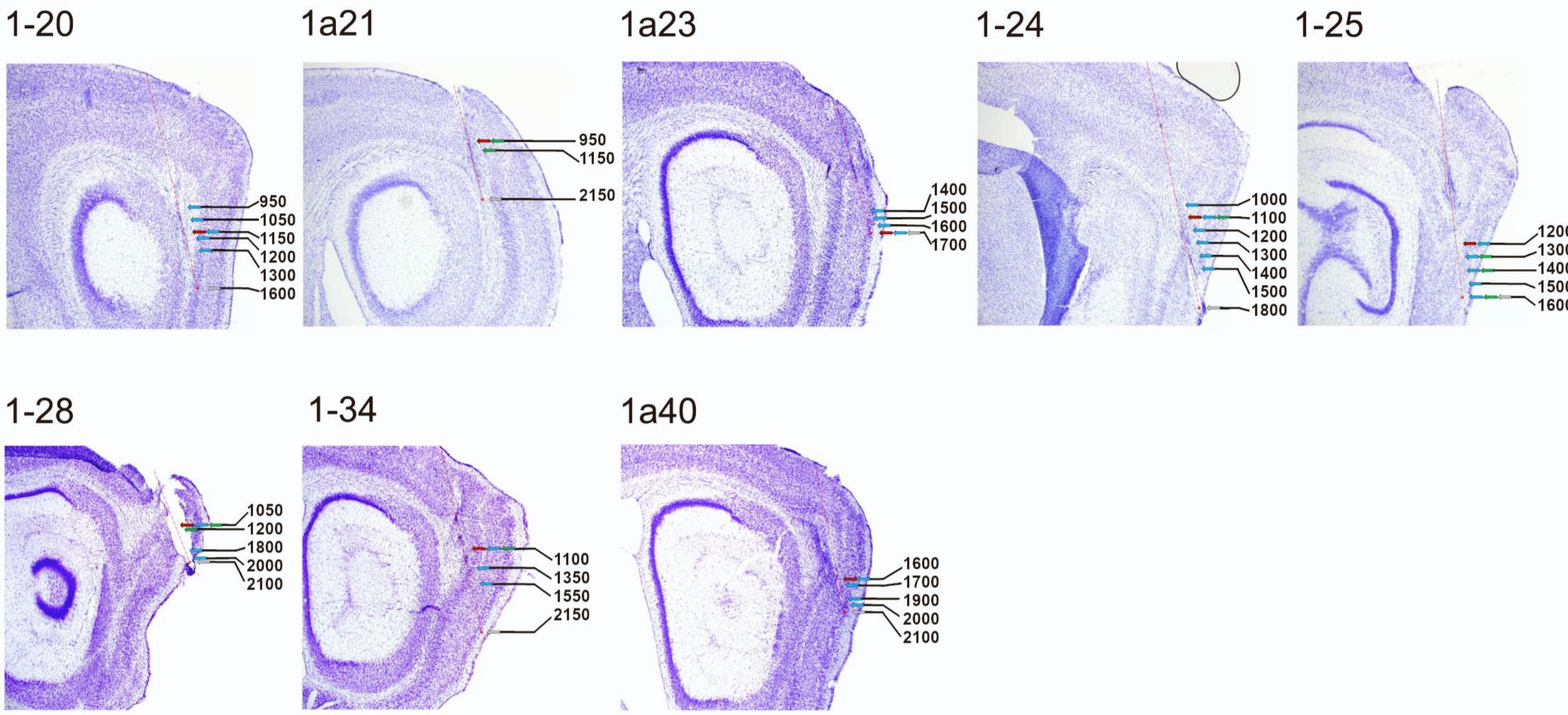

B

App ***NL-G-F*** **(APP KI)**
18-month

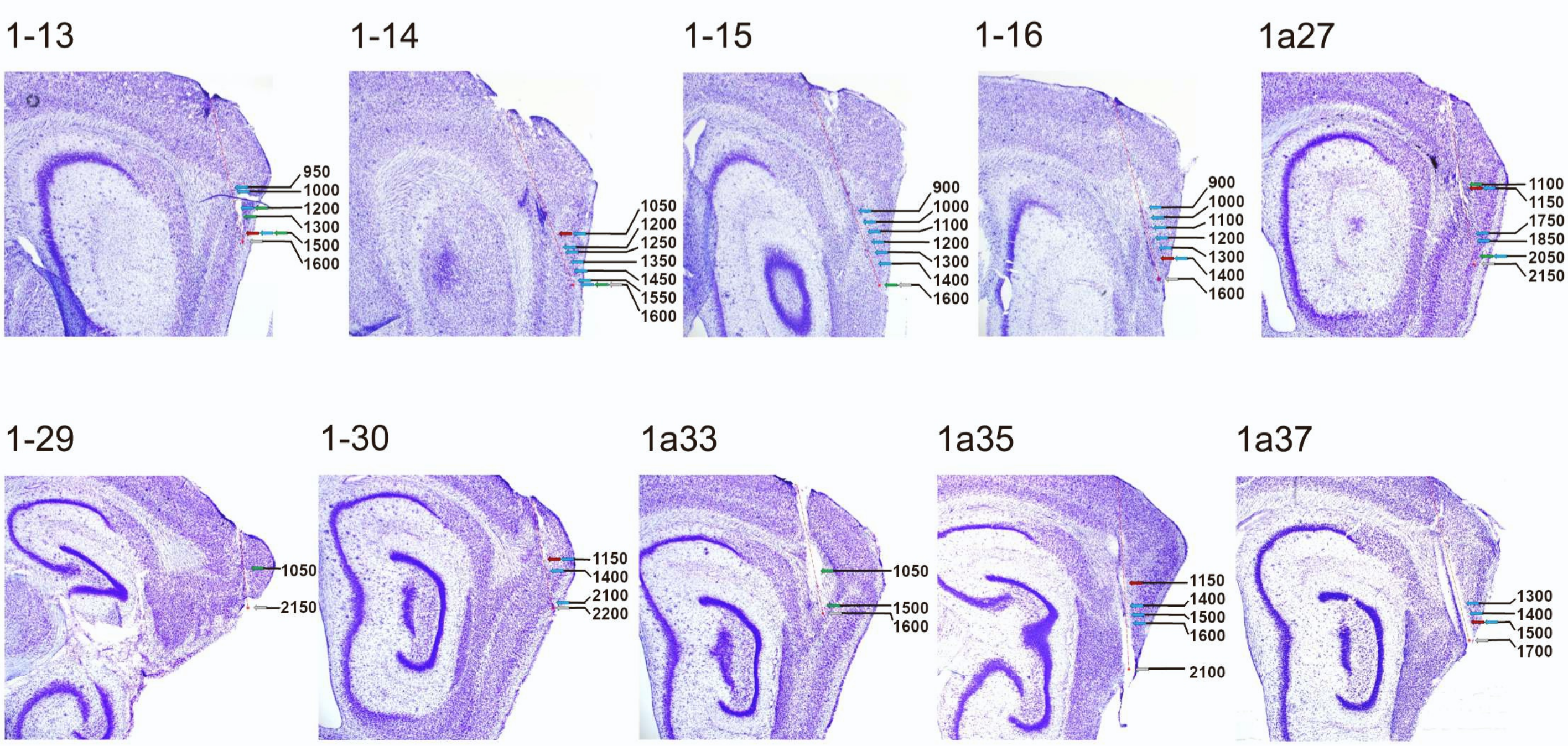

Supplemental Figure 12. Tetrode trajectories and recording positions in aged *APP* $^{NL\text{-}G\text{-}F/NL\text{-}G\text{-}F}$ mice and C57BL/6J controls. Histological verification of recording positions in 18-month C57BL/6J control mice (Total, n=8 mice; Female, n=5 mice; Male, n=3 mice) (***A***) and age-matched APP KI mice (Total, n=10 mice; Female, n=6 mice; Male, n=4 mice) (***B***) used in these studies. Brightfield images of cresyl violet-stained sagittal sections appear for each mouse and show electrode trajectories (red hashed line). Red arrows indicate recording sites where units were collected for recording block analyses (Figures 1-4, Supplemental Figures 2-3, 6-10). Blue arrows indicate recording sites where units were sampled and pooled per mouse for hyperactivity and LFP analysis (Figure 5, Supplemental Figure 11). Green arrows indicate recording sites where spatial cells examples in Figure 2 were sampled. Gray arrows represent the last recording position for each mouse.
